# Research progress on biomarkers of traumatic brain injury

**DOI:** 10.1002/ame2.70173

**Published:** 2026-03-27

**Authors:** Xuting Shen, Si Cheng, Shuyi Chen, Mengyue Wang, Tangying Li, Weicheng Xu, Yifan Zhang, Ping Yuan, Lei Shi

**Affiliations:** ^1^ Department of Clinical Laboratory Shuguang Hospital Affiliated to Shanghai University of Chinese Traditional Medicine Shanghai China; ^2^ Department of Cardio‐Pulmonary Circulation Innovation and Incubation Center (IIC), Shanghai Pulmonary Hospital, School of Medicine, Tongji University Shanghai China; ^3^ Department of Human Anatomy, Histology and Embryology School of Basic Medical Sciences, Xi'an Jiaotong University Xi'an Shaanxi China; ^4^ Institute of Neuroscience, Translational Medicine Institute Xi'an Jiaotong University Health Science Center Xi'an Shaanxi China

**Keywords:** biomarkers, diagnostics, head injury, serum biomarkers, traumatic brain injury (TBI)

## Abstract

Traumatic brain injury (TBI) is a common disorder of the nervous system and has become a leading cause of death and disability worldwide, imposing a substantial burden on patients and their social circles. Its main symptoms include dyskinesia, language acquisition difficulties, and cognitive decline. Because of its complexity and diversity, the diagnosis and treatment of TBI have consistently been key areas of focus in medical research. Traditional imaging methods, including X‐ray computed tomography (CT) scans and magnetic resonance imaging (MRI), are proficient at identifying overt structural abnormalities; however, their sensitivity in detecting subtle or hidden brain injuries is somewhat restricted. CT is insensitive to nonhemorrhagic lesions and cannot accurately evaluate the degree of injury. The detection rate of microhemorrhagic foci and specific types of TBI by MRI is low; other technologies have complicated operation and high equipment requirements. Therefore, the utilization of biological indicators, or biomarkers, in assessing and predicting the course and outcome of TBI holds immense importance in both diagnostic and prognostic evaluations. This overview reviews the research progress of biomarkers in TBI. Recognizing the significance of TBI‐related biomarkers, understanding the pertinent key molecular pathways, and staying informed about the latest advancements in treatment methods are of utmost importance. However, research on TBI biomarkers continues to face challenges. To advance the field, future efforts should focus on delving deeper into the mechanisms, refining more sensitive and tailored detection methodologies, conducting extensive clinical validations, and exploring the potential of personalized treatment plans.

## Graphical abstract

Traumatic brain injury: From primary insult to secondary neuroinflammation and degeneration.

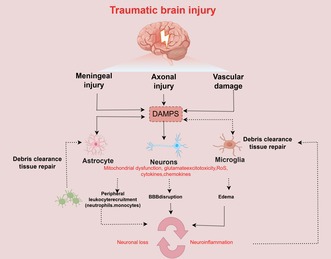

## 1 INTRODUCTION

Traumatic brain injury (TBI), frequently described as a silent prevalent condition, refers to various injuries caused by injury mechanisms and leads to a series of clinical consequences.^1^, ^2^ TBI is mainly caused by five external forces: accidental falls, violent blows, traffic accidents, sports shocks, and occupational injuries. These initial acute injuries caused by traumatic events lead to different degrees of neurological impairment by directly destroying brain tissue or inducing a secondary injury chain reaction.^3^ Therefore, TBI is considered an important disease affecting the central nervous system (CNS), which has a significant impact that cannot be ignored. Understanding its definition and classification is very important for doctors, patients, and their families because it not only damages the proper function of the brain but also brings a heavy burden to patients' families.

### 1.1 Definition and classification

According to the categorization outlined by the Centers for Disease Control and Prevention (CDC) of the United States, TBI signifies the disruption of typical brain function due to impacts, blows, jolts, or penetrating injuries to the head.^4^ The type and severity of TBI vary with individuals, external force intensity, and contact mode. This kind of injury is further subdivided into mild,^5^, ^6^ moderate,^7^, ^8^ and severe categories^9^, ^10^, ^11^ based on its severity and clinical manifestations.

#### 1.1.1 Mild TBI

Usually manifested as transient loss of consciousness, amnesia, headache, nausea, and other symptoms. Though the symptoms are relatively mild, mild TBI may still have a long‐term influence on patients' quality of life and cognitive ability.^5^, ^6^, ^12^

#### 1.1.2 Moderate TBI

Symptoms are more serious, which may include persistent headache, nausea, vomiting, confusion, or drowsiness. Patients with moderate TBI usually need longer rehabilitation and medical intervention.^7^, ^8^, ^13^

#### 1.1.3 Severe TBI

It is the most serious type, which may lead to long‐term coma, serious neurological dysfunction, and even death. Patients with severe TBI need urgent medical treatment and long‐term rehabilitation.^9^, ^10^, ^11^, ^14^

### 1.2 Damage mechanism

The pathological mechanism of TBI can be divided into two key stages: primary injury and secondary injury.^15^, ^16^ The initial injury force leads to primary injury, which causes tissue destruction and deformation in the early stage after injury.^17^ The secondary damage is caused by various molecular and pathophysiological reactions, which eventually lead to the death of neurons. This process involves a variety of molecular and pathophysiological reactions, such as inflammatory reaction, oxidative stress, apoptosis, and so on, which further aggravate the death of neurons and the damage of brain tissue.^18^, ^19^ Cell damage and death trigger the release of ions, molecules, and proteins to the outside of the cell, a process named as the damage‐related molecular model (DAMP).^20^ Among them, neuroinflammation is the key pathological process of secondary TBI, which is triggered by the activation of microglia by DAMPs (such as reactive oxygen species [ROS], cell debris, and heme),^21^, ^22^ leading to adverse changes in astrocytes. Regulating microglial activation and downstream inflammatory signals is a potential method to treat secondary TBI and prevent long‐term cognitive and emotional disorders.^22^

### 1.3 Symptoms and signs

The variety of symptoms and indicators associated with TBI may encompass loss of consciousness, memory loss, nausea, dizziness, headaches, and a decline in cognitive functions. In addition, patients may have structural brain injuries, such as cerebral hemorrhage, brain contusion, and laceration, which may be confirmed by imaging examination. Other neurological symptoms, such as paresthesia and dyskinesia, may also appear after TBI.^23^

### 1.4 Incidence and prevalence rate of TBI

In 2017, a global report in *The Lancet Neurology* warned that TBI, as the highest incidence disease of the nervous system, caused 50–60 million cases and 400 billion dollars in losses every year, and it is expected that by 2030 it will become one of the three main causes of injury, disability, and death.^3^ The global disease burden data in 2019 show that TBI poses a major challenge to global health. There are 27.16 million new cases and 48.99 million epidemic cases of TBI.^24^ According to the report released by the World Health Organization in 2021, TBI has become a severe public health challenge, resulting in more than 1 million cases worldwide every year, and it occupies a dominant position in the disability factors of young people.^25^ The annual economic loss caused by brain injury promotes the innovation demand for TBI monitoring technology in academic circles. At present, the research focuses on developing new evaluation methods with clinical practicability, safety, and cost‐effectiveness.^26^

### 1.5 The pathophysiological mechanism

**FIGURE 1:**
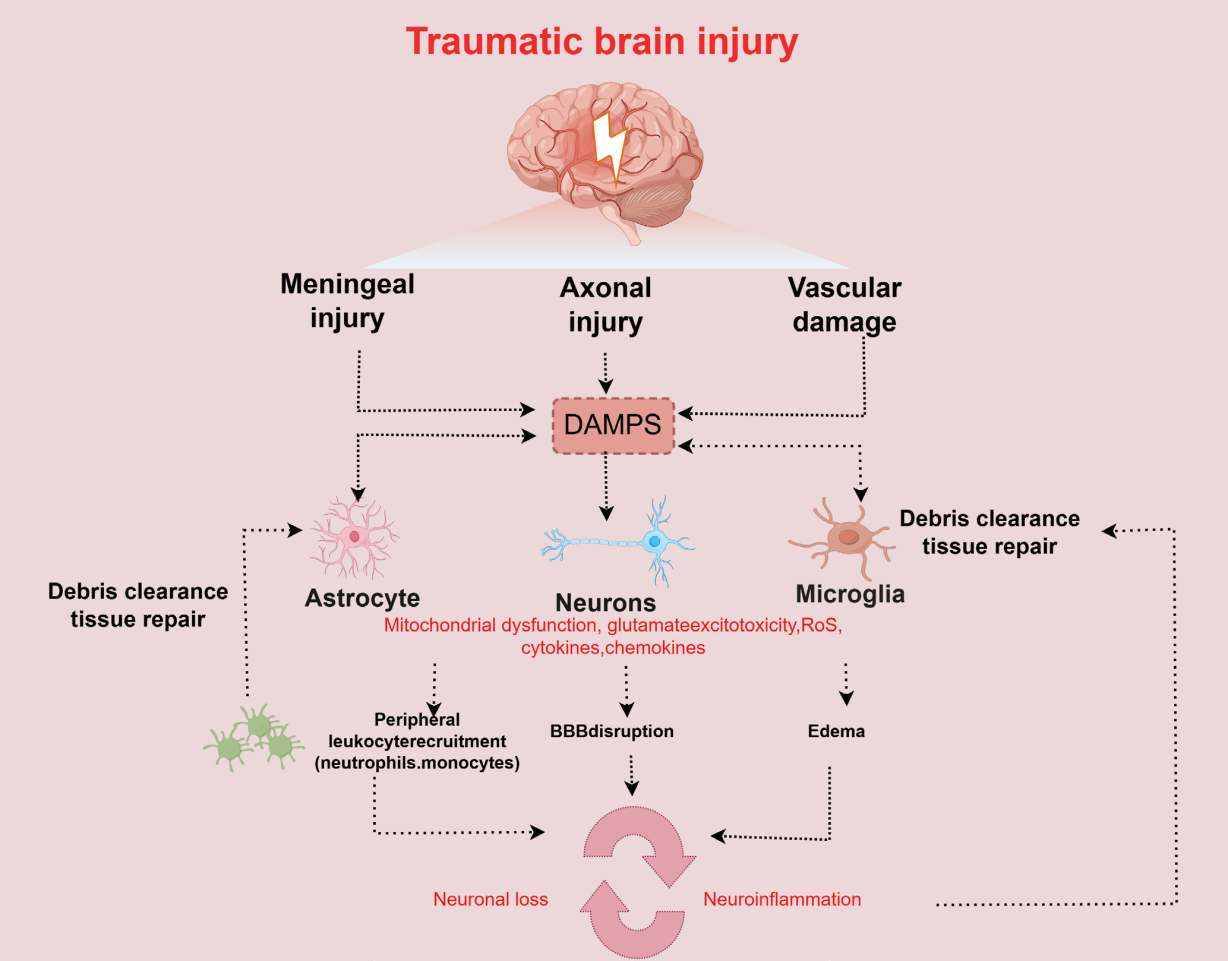
Pathophysiology of traumatic brain injury (TBI). DAMPS, damage‐associated molecular patterns.

TBI has a complicated pathogenesis, which mainly includes two key stages: primary injury and secondary injury. Exploring and clarifying the precise mechanism of primary and secondary pathology related to TBI are key to developing effective treatment strategies for TBI (Figure 1).^27^, ^28^

#### 1.5.1 Primary stage

It is characterized by mechanical trauma. In clinical TBI cases and experimental TBI models, the starting point of traumatic injury is the mechanical damage suffered by endothelial cells, neurons, and glial cells.^29^, ^30^ The subsequent injuries encompass contusions, damage to the vasculature, hemorrhages, changes in cerebral blood circulation, and modifications in the permeability of the blood–brain barrier (BBB), along with disruptions in metabolic processes. TBI will lead to the destruction of the BBB and subsequently stimulate the release of macrophages, neutrophils, and lymphocytes within the affected region. The researchers found that TBI led to an increase in immune cells that have migrated from the bloodstream into the substance of the brain, which release inflammatory mediators to draw immune cells and glia to the site of injury. Furthermore, the activation of microglia also plays a key role, and its synapses form a defense barrier between health and injury.^31^, ^32^ These secondary injuries involve intricate, multifaceted biochemical reactions that commence shortly after the initial mechanical trauma and persist for extended periods, ranging from days to months and even years postinjury. They contribute significantly to the exacerbation of neuroinflammation, neurodegeneration, and the manifestation of neurological impairments.^33^ These immediate injuries exert detrimental effects on cerebral parenchyma, disrupt the cerebrovascular barrier, and impact neurons, glial cells, and the cerebrovascular system.^34^

#### 1.5.2 Secondary stage

**FIGURE 2:**
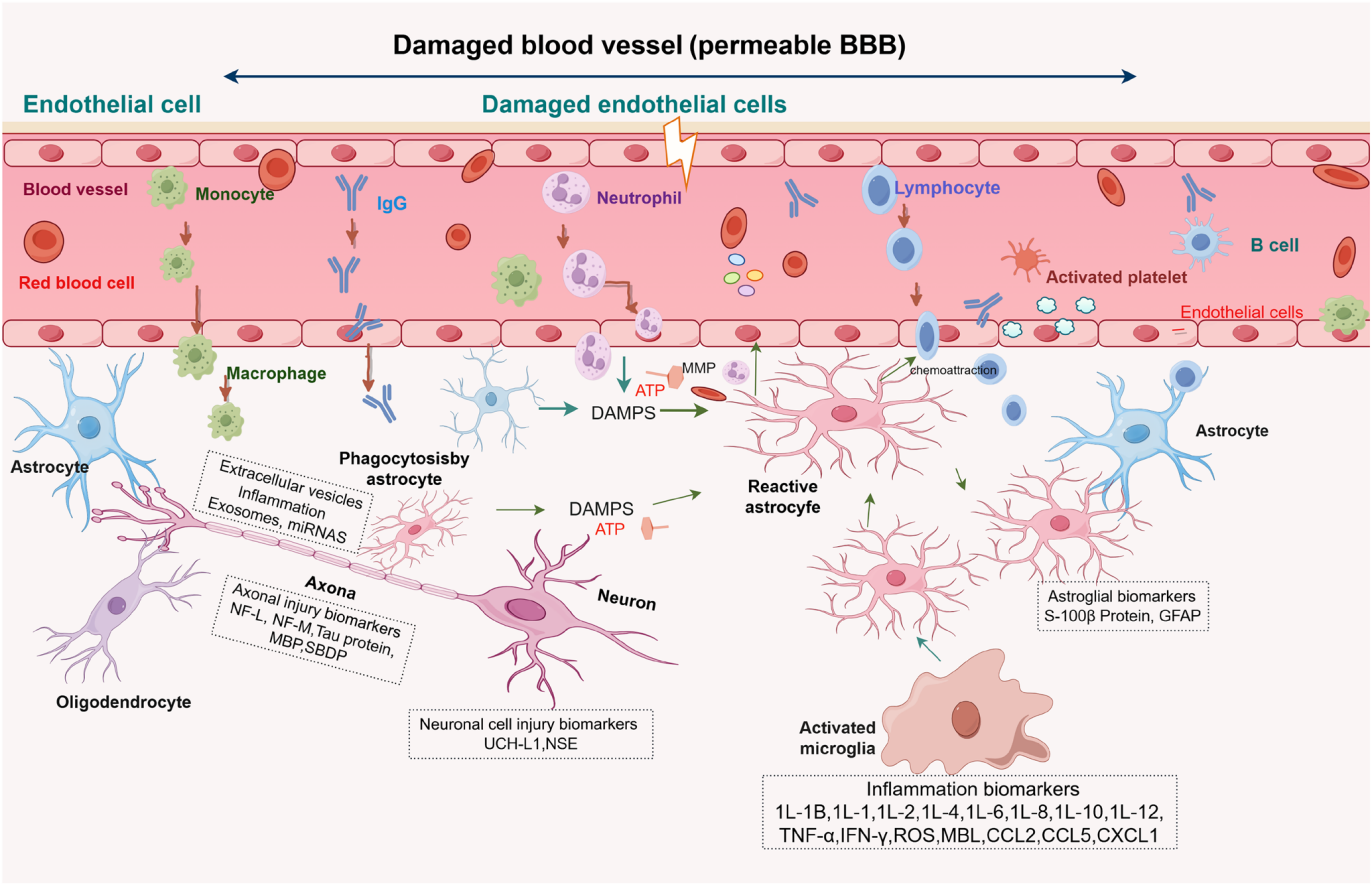
Traumatic brain injury (TBI) can trigger a variety of signal pathways and then induce corresponding changes in astrocytes. ATP, adenosine triphosphate; BBB, blood–brain barrier; CCL2, C‐C motif chemokine ligand 2; CCL5, C‐C motif chemokine ligand 5; CXCL1, C‐X‐C motif chemokine ligand 1; DAMPs, damage‐associated molecular patterns; GFAP, glial fibrillary acidic protein; IFN‐γ, interferon‐gamma; IL‐1, interleukin‐1; IL‐10, interleukin‐10; IL‐12, interleukin‐12; IL‐1β, interleukin‐1 Beta; IL‐2, interleukin‐2; IL‐4, interleukin‐4; IL‐6, interleukin‐6; IL‐8, interleukin‐8; MBL, mannose‐binding lectin; MMP, matrix metalloproteinase; NF‐L, neurofilament light chain; NF‐M, neurofilament medium chain; ROS, reactive oxygen species; TNF‐α, tumor necrosis factor‐alpha.

Following the initial phase, a cascade of biochemical alterations ensues, involving intricate interactions among neurotransmitters, biochemical mediators, cytokines, and genetic modifications, ultimately causing tissue deterioration. The progression of secondary injuries entails persistent alterations in the flow of intracellular ions, particularly calcium (Ca^2+^), sodium (Na^+^), and potassium (K^+^), as well as the release of neurotoxic transmitters such as glutamate.^35^ Furthermore, proteins from the cytoplasm and nucleus, which are released from injured cells, function as damage‐associated DAMPs; these DAMPs serve as potent activators of both central and systemic immune responses.^36^ Immune cells (macrophages, neutrophils, lymphocytes) infiltrate around damaged nerve cells to remove debris and myelin sheath. At the same time, the factors released by these cells and microglia activate astrocytes, which further promote the increase in phagocytes and the activation of microglia, and lead to fragility of the BBB (through MMP release), thus attracting more circulating immune cells to the lesion site (Figure 2).^37^ These changes will trigger brain events such as oxygen deficiency, oxidative damage, cellular necrosis, cell suicide, and the development of prolonged inflammation.^38^, ^39^, ^40^ Secondary injuries are mostly reversible,^41^, ^42^ including excitotoxicity,^43^ production of free radicals and peroxidation of lipids,^44^, ^45^ astrocyte swelling and loss,^46^ impairment of mitochondrial function, deterioration of axons, and neuronal cell death. Furthermore, microglia undergo proliferation, shift toward the damaged region, and produce cytokines.^47^ When microglia are overactivated or react, they will release a series of harmful substances, including oxidative metabolites (such as nitric oxide and ROS) and various proinflammatory cytokines, such as tumor necrosis factor α (TNF‐α), interleukin‐1β (IL‐1β), and interferon γ (IFN‐γ).^48^ They contribute to the restoration and rejuvenation of neural tissue within the CNS. However, they can also cause harm to neurons and glial cells by exhibiting neurotoxic effects.^49^ These substances hurt neurons, further aggravating brain damage. In addition, the generation of inflammatory cytokines and their associated factors will continue to stimulate astrocytes and glial cells, leading to scar formation. In this process, the activation signs of astrocytes include the development of intermediate filaments (such as glial fibrillary acidic protein [GFAP] and vimentin), the increase in cell accumulation, and cell swelling.^50^

There are various pathophysiological changes in TBI,^51^ which vary based on the site, nature, and magnitude of the injury.^52^, ^53^ Mild injury may lead to temporary discomfort and symptoms, whereas moderate‐to‐severe injuries can cause significant primary damage and subsequent secondary injuries, encompassing diffuse axonal injury (DAI), hematoma, intracerebral hemorrhage, extensive brain tissue destruction, and potentially fatal outcomes.^54^ Primary injury is irreversible, whereas secondary injury may be prevented by appropriate surgical intervention and intensive treatment measures.^38^, ^55^, ^56^, ^57^, ^58^, ^59^, ^60^ However, TBI has brought long‐term challenges to researchers because of its complex pathophysiology, diverse clinical manifestations, and delicate balance needed for treatment. Therefore, it is crucial to identify and quantify the various subtypes of TBI in a noninvasive and dependable manner to devise a therapeutically appropriate treatment strategy.^61^

### 1.6 The harm and influence of TBI

TBI can lead to impairments in the domains of cognition, attention, anxiety states, aggressive tendencies, depressive disorders, and alterations in personality. Additionally, it is postulated to increase the likelihood of developing certain conditions, such as Parkinson's disease and Alzheimer's disease (AD),^54^ which are defined by multilevel medical,^62^ neuropsychiatric,^63^ cognitive,^64^ emotional,^65^ and psychosocial sequelae.^66^ If the consequences of TBI are left untreated, these conditions may not only exert prolonged detrimental effects on the health of affected individuals but also impair their health‐related quality of life^67^ and the well‐being of their family members.^68^, ^69^ Acknowledging the diverse nature of TBIs and their changing clinical course, refining outcome forecasts may entail the integration of new data over time or the inclusion of factors that forecast treatment responsiveness.^70^ The annual economic burden imposed by brain injuries motivates researchers to discover effective, safe, and cost‐efficient methods for monitoring TBI.^26^ The varied and intricate characteristics of TBI, coupled with a limited understanding of its underlying pathophysiology, pose significant challenges in developing effective therapeutic strategies. Because of its heterogeneity and complexity, and the lack of understanding of the underlying pathophysiological mechanism, TBI faces great challenges in developing effective treatment methods. At present, there are still obvious obstacles in establishing clear and effective treatment strategies, diagnostic techniques, prognosis evaluation systems, and optimizing patient stratification and feature description to improve management and treatment.^71^, ^72^ Therefore, there is an urgent need to develop more accurate and efficient diagnostic methods for TBI to promote overall improvements in its treatment and management.

## 2 BIOMARKERS OF TBI

### 2.1 Definition of biomarkers

Biomarkers refer to the internal indicators of organisms that can be measured at the molecular or cellular level. They can reflect the stress, dysfunction, or injury of organisms and provide information about the injury mechanism.^28^ In the diagnosis of TBI, biomarkers are a key tool to quickly identify injury; evaluate the condition; reflect the biological process, disease state, or treatment effect; and help doctors accurately identify the degree of injury, predict the prognosis, and monitor the curative effect.^73^ As per the American Food and Drug Administration's (FDA) definition, biological markers serve as quantifiable indicators of normal physiological functions, disease processes, or reactions to particular exposures or treatments (inclusive of therapeutic measures).^74^, ^75^, ^76^, ^77^ These biomarkers are not only easy to measure but also have clinical significance and are used as signs of normal or disease processes.^78^, ^79^, ^80^ In the context of TBI, biomarker research focuses on their potential use as a diagnostic, prognostic, and tracking instrument for patients with brain injuries.^81^, ^82^ Through the integration of biomarker analysis, clinical evaluation, and imaging methodologies, we can accurately determine TBI severity, predict outcomes, and assess responses to treatment.^83^ In the context of TBI, biomarkers can be used not only as diagnostic markers of injury severity but also as monitoring markers of treatment response and as indicators of posttraumatic outcomes.^84^

### 2.2 Characteristics of biomarkers of TBI

**FIGURE 3:**
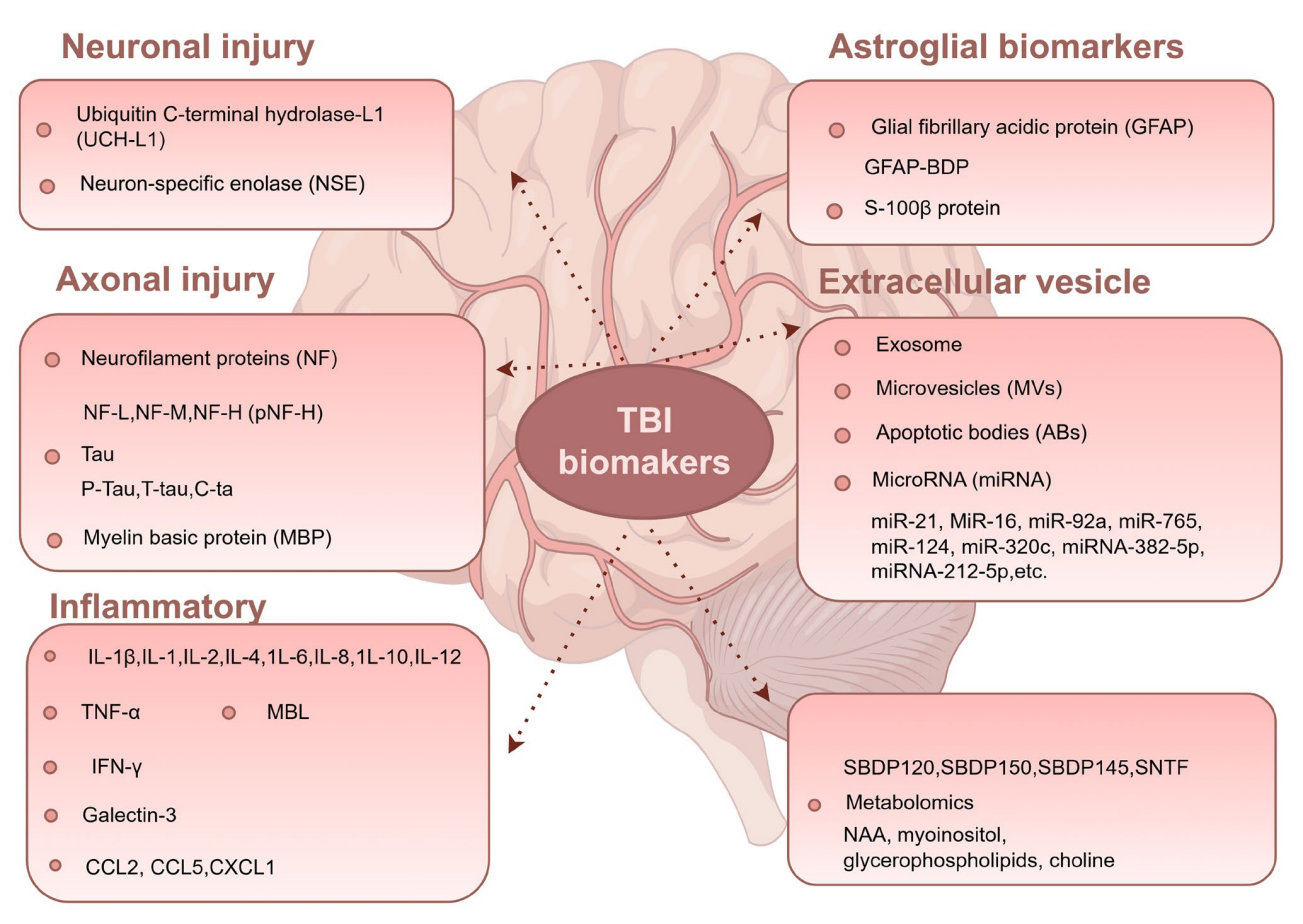
Classification of traumatic brain injury (TBI) biomarkers. CCL2, C‐C motif chemokine ligand 2‐5; CCL5, C‐C motif chemokine ligand 5‐5; CXCL1, C‐X‐C motif chemokine ligand 1‐5; IFN‐γ, interferon gamma‐1‐9; IL‐1, interleukin‐1; IL‐10, interleukin‐10; IL‐12, interleukin‐12; IL‐1β, interleukin‐1 Beta; IL‐2, interleukin‐2; IL‐4, interleukin‐4; IL‐6, interleukin‐6; IL‐8, interleukin‐8; MBL, mannose‐binding lectin; SBDP120, spectrin breakdown product 120 kDa; SBDP145, spectrin breakdown product 145 kDa; SBDP150, spectrin breakdown product 150 kDa; SNTF, S‐nitrosylated transferrin; TNF‐α, tumor necrosis factor‐alpha.

The attributes of TBI biomarkers include high specificity to brain tissue, high sensitivity, prompt release postinjury, and a duration that closely correlates with injury severity and clinical outcomes.^28^, ^85^ From a clinical perspective, sensitivity measures a biomarker's ability to identify patients who have a specific disease or condition, whereas specificity evaluates its capacity to differentiate between patients who do not have the disease.^86^ Studies conducted over the past few decades have revealed a growing interest in investigating biomarkers of brain injury as potential instruments for prognostic assessment.^87^, ^88^ This review focuses on several key biomarkers of brain injury: neuronal injury (neuron‐specific enolase [NSE], ubiquitin C‐terminal hydrolase‐L1 [UCH‐L1]), astroglial injury (GFAP, S‐100β), axonal injury (neurofilament [NF] proteins, tau protein, and myelin basic protein [MBP]), inflammatory (1L‐6, 1L‐10, TNF‐α), extracellular vesicles(exosomes and microRNA [miRNA]), and other emerging biomarkers, such as αII‐spectrin breakdown products and metabolomics, chosen for their established status as notable candidate biomarkers associated with TBI pathophysiology,^89^ and they exhibit temporal profiles as depicted in Figure 3.^90^

### 2.3 Research status of biomarkers of TBI

TBI assessment in clinical practice is a multifaceted process that typically encompasses monitoring of acute injuries, neuropsychological evaluations, brain imaging studies, and meticulous documentation of signs and symptoms. These evaluation methods together form the basis of TBI diagnosis, aiming at comprehensively understanding the degree of injury, neurological function, and prognosis of patients.^91^, ^92^, ^93^ The elevated morbidity and mortality rates linked to TBI are partly attributed to the restricted diagnostic and therapeutic tools that fail to adequately represent the variety of TBI‐related injuries, subsequently hindering the progression of innovative treatments.^28^, ^94^, ^95^

In clinical settings, severe TBI (sTBI) is commonly classified based on the initial Glasgow Coma Scale (GCS) score at hospital admission, whereas the Glasgow Outcome Scale (GOS) is used to assess long‐term clinical outcomes.^96^ GOS spans a range from 1, indicating death, to 8, signifying full recovery. Contemporary models incorporate the GCS as one of several factors in predicting patient recovery outcomes.^97^ When used in combination with CT imaging, the GCS serves as a tool for assessing the severity of TBI and forecasting clinical outcomes for patients.^98^ However, these classifications are subject to debate owing to their insufficient description of the variability associated with TBI.^99^ For instance, the utility of the GCS score in diagnosing mild TBI is constrained, especially in cases involving polytrauma, alcohol abuse, use of sedatives, and psychological distress.^100^ After the development of GCS^101^ and GOS,^102^ it was found that confident predictions could be made after 24 h following the injury but were difficult to establish on admission.^103^ The GCS evaluates consciousness and offers prognostic insights; however, it does not elucidate the underlying pathologies that could be the focus of treatment.^104^, ^105^ Investigations spanning the past two decades have indicated that the GCS alone may not be sufficient for diagnosing or predicting the prognosis of TBI, especially in cases of mild injury.^106^ Although GCS is still the basic tool for TBI severity grading, the limitation of its single dimension prompted the National Academy of Sciences and NINDS to launch the CBI‐M multidimensional evaluation framework in 2024—integrating clinical dimensions (refining GCS subitem score + independent pupil response record), biomarkers (such as GFAP/UCH‐L1), imaging characteristics and modification factors (comorbidity/age/injury mechanism, etc.). The framework was developed by six expert groups (including the Clinical/Symptom Working Group [CSWG]) based on an evidence‐based consensus, emphasizing the standardized record of loss of consciousness, posttraumatic amnesia, and 14‐day dynamic monitoring, and incorporating biopsychosocial variables to optimize individualized diagnosis and treatment; however, its integration efficiency with clinical practice needs to be verified further.^107^

In the majority of cases, TBI is identified through a comprehensive neurological assessment of the patient, coupled with readily available imaging modalities like CT and MRI in a clinical setting.^108^ MRI is commonly used in clinical settings and has gained popularity for quantifying brain damage, especially in instances of widespread TBI. The recently introduced diffusion tensor imaging method can sense detailed alterations in white matter microstructure and holds significant promise for assessing axonal damage.^109^ These variables have been incorporated into forecasting models, achieving some success in predicting outcomes but with certain constraints.^110^, ^111^ Imaging modalities do not constitute a conclusive diagnostic tool for TBI, as they are unable to identify changes in a substantial number of patients with mild‐to‐moderate traumas.^112^, ^113^, ^114^ DT‐MRI is viewed as a potential diagnostic aid for TBI, attributed to its ability to concentrate on axonal structures. Nevertheless, the existing literature on the detection of acute mild TBI (mTBI) shows a degree of inconsistency.^115^, ^116^ Due to their limited sensitivity and the absence of hemorrhage, standard imaging methods such as CT and MRI often fail to detect lesions resulting from traumatic injuries, posing difficulties in characterizing and assessing the outcomes of such injuries. Although severe TBI may have a clearer prognosis, identifying mild and moderate cases of TBI proves to be a considerable challenge.^117^

During the initial assessment phase for patients suffering from TBI, there are potential inaccuracies in neurological severity, and in some subgroups of patients, the severity of injury may be overestimated, which means based on the results of a preliminary evaluation, some patients' actual conditions may be lighter than expected, which may affect their treatment strategies and rehabilitation plans.^118^ Traditional CT scans and standard MRIs underestimate the degree of white matter damage that occurs following TBI.^115^, ^119^ It is crucial to note the concerns regarding the potential consequences of radiation exposure from CT scans,^120^, ^121^ particularly in the context of repeated imaging for reassessment of existing injuries or recurrent trauma.^122^ Furthermore, CT scans expose patients to potentially harmful ionizing radiation and increase healthcare costs.^123^ Therefore, an accurate diagnosis that complements medical and radiological evaluations is essential. Biomolecular markers in bodily fluids are viewed as an objective and prompt means of confirming the identification of TBI during extended periods following the injury.^124^ Consequently, there has been a surge in the quest for novel or supplementary risk assessment tools and biomarkers. In conclusion, the current diagnostic methods of TBI have many limitations, and biomarkers, as an objective and sensitive indicator, are gaining increasing significance for diagnosing TBI.

The evolution of biomarker development for the detection of TBI from body fluids is a multidisciplinary research field, aiming at early diagnosis of TBI, assessment of illness, prediction of prognosis, and monitoring of treatment response through biomarkers in body fluids. Biomarkers indicative of brain injury can be detected in cerebrospinal fluid (CSF) and blood immediately following TBI.^125^ These biomarkers are not intended for use as solitary diagnostic or prognostic tools, but rather, they are meant to complement clinical and radiological information in the comprehensive process of making a clinical decision. Within the initial 24 h after a TBI, the presence of cell death facilitates the release of brain proteins and their metabolic by‐products from damaged cells into biological fluids.^28^, ^126^, ^127^, ^128^ Biomarkers can primarily be analyzed in either the CSF or peripheral blood.^129^ The CSF, which is in immediate contact with the extracellular environment of the brain, mirrors the biochemical alterations occurring within the organ.^130^ Given these considerations, CSF can be seen as an optimal source of biomarkers for detecting brain injury. However, the collection of CSF is relatively complicated and has certain risks, which limit its wide application in the clinic. Blood, noted for its ease of accessibility, simplicity in handling, relatively standardized samples, and the availability of ample normative information, serves as a favored medium for biomarker discovery. Nevertheless, the fact that blood interacts with various tissues and organs may complicate the task of determining the source of a particular protein and introduce challenges in terms of sensitivity and dynamic range.^131^ The BBB, a complex vascular construct comprising multiple cell types, functions as a selective permeability barrier, restricting the passage of most substances from the bloodstream into the brain, thereby preserving brain homeostasis.^132^, ^133^ After TBI and disruption of the BBB, brain‐derived proteins that are liberated from compromised neurons enter the circulation, potentially eliciting an immune reaction. Immediately after TBI, indicators of brain injury, which serve as biomarkers, can be detected in both CSF and blood.^125^ The serum albumin ratio serves as a conventional biomarker for assessing BBB function.^134^ Most of the albumin present in the CSF originates from the blood, crossing the BBB.^135^

Light NF and neurofilament heavy (NF‐H) serve as markers of damage to thick myelinated axons and myelinated nerve fibers. In contrast, total tau (T‐tau) serves as evidence of the deterioration of thin, nonmyelinated nerve fibers. Damage to astroglial cells may trigger the release of S‐100β and GFAP within the extracellular environment, potentially elevating S‐100β concentrations in both CSF and blood. Astrocytic activation and neuroinflammation postinjury can trigger an increase in the production of interleukins and cytokines. The amounts of these biomarker compounds in bodily fluids, assessed either individually or in combination, may indicate injury severity and predict a positive outcome on CT scans for patients with TBIs.^136^

## 3 STANDARD FOR INCLUSION AND EXCLUSION OF TBI BIOMARKERS

TBI biomarkers are important tools for the diagnosis and prognosis of TBI. For TBI biomarkers to have clinical value, they should ideally possess as many of the following attributes as possible.

### 3.1 Types of research

In terms of screening criteria, it is required that the selected documents must be original research reviewed by peers, adopt qualitative research methods, and provide analyzable original data. The research on mixed methods, including both qualitative and quantitative data, is also included. In terms of research types, different levels of evidence, such as randomized controlled trials (RCTs), cohort studies, and case–control studies, should be clearly included. For the field of TBI biomarkers, both exploratory research (finding new markers) and confirmatory research (evaluating the clinical efficacy of known markers) should be included.

### 3.2 Population characteristics

Classification of injury severity (based on GCS‐P scoring system combined with pupil reactivity score [PRS]).

Age range (biomarker differences between children, adults, and the elderly). Acute and chronic stages (acute stage <7 days, subacute stage 7 days to 6 months, chronic stage ≥6 months).

### 3.3 Eliminate standard key concerns

Study on other nervous system diseases (such as AD and Parkinson's disease). Methodological description is incomplete (key parameters such as detection limit and recovery rate are not reported).

### 3.4 Data extraction contains the following core fields

Basic research information (author, year, country).

Study design (type, sample size, follow‐up time).

Patient characteristics (age, sex, TBI severity).

Details of biomarkers (name, sampling time, detection method).

Main results (accuracy of diagnosis/prognosis, association with clinical outcome).

Research quality score (such as QUADAS‐2 tool).

## 4 RESEARCH PROGRESS OF TBI BIOMARKERS

### 4.1 Neuron‐specific enolase

#### 4.1.1 Introduction of NSE

NSE, a dimeric protein with a molecular weight of 78 kDa composed of the γ‐isoform, is predominantly localized in neuronal cytoplasm and has a vital part in facilitating axonal transport.^137^, ^138^ It is typically not secreted outside the cell by healthy neurons; however, when the axon is damaged, NSE levels may be upregulated to maintain homeostasis within the body,^139^ and it will be detected in the cytoplasm around the broken axon due to leakage.^140^ Moore and McGregor first characterized NSE in 1965, and it is an important biomarker for evaluating neuronal damage.^141^

#### 4.1.2 The role of NSE in TBI

In the aftermath of TBI, pathological conditions can lead to the passive release of NSE into the extracellular space during neuronal cell damage^142^ and subsequently permeate into the CSF and blood serum after neuronal cell death caused by traumatic injury.^143^, ^144^ This mechanism renders serum NSE an excellent candidate biomarker for monitoring ongoing injury post‐TBI.^145^ It has been investigated as a TBI biomarker in numerous pediatric^145^, ^146^, ^147^, ^148^, ^149^, ^150^, ^151^ and clinical trials involving adult subjects^152^ and preclinical studies in adult models.^153^ In this context, extracellular NSE serves as an indicator of neuronal cell injury^154^ and is utilized in guidelines for managing cardiac arrest.^155^ Due to its prevalence and location within biofluids, NSE is anticipated to demonstrate a high degree of accuracy and reliability in identifying axonal damage.^143^, ^156^ Consequently, NSE shows promising potential utility as a prognostic marker for sustained periods and as a monitoring tool in therapeutic interventions within intensive neurology care environments.^157^, ^158^

For numerous TBI patients, the first peak occurs within 6 h after injury, and the concentration is related to the severity of prognosis, followed by a continuous high concentration with a slow decline rate and the secondary peak at 72 h.^159^ Furthermore, in individuals with widespread brain damage and increasingly severe secondary brain injuries, NSE values remain persistently high. Consequently, NSE concentrations not only reflect the extent of initial brain injury but also signify the development of subsequent injuries. Therefore, NSE holds significant potential usefulness as a chronic prognostic tool and a prognostic indicator for therapeutic outcomes in high‐acuity neurological care.^157^, ^158^ Initially, measurements of NSE were analyzed in serum and ventricular CSF samples obtained from patients suffering from critical brain trauma and coma, underscoring its potential as a key marker of neuronal injury.^160^ For both adults and children, NSE concentrations in ventricular CSF correlate with fatality rates post‐TBI and with additional severity assessments for TBI, like the GCS and GOS.^161^ Notably, NSE is the exclusive marker for directly assessing functional neuronal harm present in the cytoplasmic compartment of neurons. NSE has been reported to appear rapidly in the serum following head injury.^162^ It can increase within minutes or hours after TBI and typically declines 1‐week posttrauma.^90^, ^145^

The first study was conducted in 2002 to measure the level of NSE within the CSF of pediatric patients, encompassing both infants and children who have sustained critical TBI.^143^ In another study focusing on pediatric patients, the effectiveness of NSE as a biomarker for predicting the prognosis of TBI in children is thoroughly evaluated.^148^ The concentrations of NSE in CSF collected through lumbar puncture have surfaced as a prospective means of detecting TBI in pediatric patients.^143^, ^163^, ^164^, ^165^, ^166^ Several research endeavors have suggested an increase in blood NSE levels after TBI, implying its potential clinical importance as an indicator for this type of trauma.^144^, ^163^, ^167^, ^168^ With a serum half‐life ranging from 24 to 72 h, NSE exhibits a longer temporal profile compared to S100‐β, UCH‐L1, and GFAP,^144^, ^146^, ^152^ rendering it a more favorable prognostic biomarker for predicting outcomes and monitoring the effects of treatment.

#### 4.1.3 The challenge and future direction of NSE research

##### The challenge of clinical application

Although the study of NSE in TBI has attracted much attention, many studies have shown that measuring NSE concentrations in biological fluids, including serum and CSF,^169^, ^170^, ^171^, ^172^ its results have not been widely used in clinical practice. This variability may stem from the differing critical NSE values that predict adverse prognoses across studies, as well as the multitude of factors (such as sample hemolysis and inflammation) that can influence NSE levels. Initially, NSE demonstrated the potential to serve as an indicator of brain damage, attributed to its apparent specificity to neuronal tissue. However, subsequent studies uncovered the presence of NSE within erythrocytes and thrombocytes, reducing its utility as a diagnostic marker because of the potential for contamination from lysis of red blood cells in blood specimens.^173^ Increased levels of NSE have been observed in diverse traumatic situations, both involving and not involving TBI, which restrict its capacity to accurately differentiate the severity of brain injury.^174^

##### Methodological challenge

At present, the enzyme‐linked immunosorbent assay (ELISA) used in most studies may not be suitable for evaluating NSE as a sign of CNS trauma, because it does not distinguish between heterodimers of enolase and evaluate the enzyme activity of this group of proteins. Therefore, it is necessary to improve the methodology for evaluating NSE as a biomarker.^175^

##### Future research direction

Future research should further explore more accurate and sensitive NSE detection methods and clarify their clinical application value in different types of TBI. At the same time, it is necessary to further study the combined application of NSE and other biomarkers (such as S100‐β, IL‐6, etc.) to improve the accuracy of prognosis evaluation of TBI patients.

### 4.2 Ubiquitin C‐terminal hydrolase‐L1

#### 4.2.1 Introduction of UCH‐L1

During the first few years of the 1980s, it was initially reported that UCH‐L1 is a protein unique to the human brain, possessing a molecular weight of approximately 27 kDa, as identified through high‐definition two‐dimensional PAGE using polyacrylamide. Subsequently, in 2006, it was identified as a potential marker of TBI for the first time by playing the role of protein omics analysis on the rat model.^176^ In recent years, due to the continual advancement of proteinomics and other sophisticated technologies, UCH‐L1 has garnered attention from researchers as a novel potential marker of brain injury, attributed to its neuron‐specific and concentrated distribution within neuronal cell bodies.^177^, ^178^, ^179^, ^180^

UCH‐L1 is a protein that exhibits high specificity for neurons, is abundant in the cytoplasm of these cells, and comprises around 5% of the total protein content in neurons.^176^, ^181^, ^182^ Although trace amounts may be found in cells beyond the nervous system, their expression is predominantly concentrated in the brain, demonstrating a high degree of neuronal specificity.^182^, ^183^ UCH‐L1 is a vital component of protein metabolism, assisting in the removal of damaged and improperly structured proteins across healthy and diseased conditions during the ubiquitination stage of the proteasome pathway.^184^, ^185^, ^186^, ^187^ Its function gives UCH‐L1 a key role in controlling proteasome pathways and regulating brain protein metabolism.^188^ At the same time, UCH‐L1 helps eliminate abnormal neuronal proteins under both physiological and pathological conditions, which is essential for maintaining normal neuronal function.^83^, ^189^ Given the prevalent occurrence of UCH‐L1 within neurons, its strong specificity to the brain, and its key role in protein metabolism, researchers think of it as a biomarker of TBI.^182^ In recent protein omics research, UCH‐L1 was identified as one of the few candidates for TBI biomarkers because of its unique characteristics and potential.^190^, ^191^

#### 4.2.2 The role of UCH‐L1 in TBI

##### Evaluate the extent of the illness and facilitate prompt diagnosis

In‐depth discussions were held regarding fluctuations in plasma CSF concentrations of UCH‐L1 and posttraumatic TBI, and their correlation with trauma severity and clinical results. The study demonstrated a significant elevation of UCH‐L1 concentrations in both blood serum and CSF during the initial 6–24 h period after TBI.^167^ This surge is primarily attributed to the disruption in the BBB integrity following moderate‐to‐critical TBI, leading to the liberation of UCH‐L1 from neuronal cytoplasm into the bloodstream.^191^, ^192^, ^193^ Further examination indicated a strong link between post‐TBI serum UCH‐L1 levels and the severity of the injury, along with clinical results. Notably, UCH‐L1 concentrations correlate with the GCS score upon admission and the injury severity depicted using CT scans.^28^, ^194^ Additionally, elevated levels of UCH‐L1 in both serum and CSF within the first 6 h of postinjury correlate with TBI severity and are associated with mortality within 3 months.^183^ Findings from rat models corroborate these observations, showing a marked elevation of UCH‐L1 concentrations in CSF and serum after a controlled cortical impact TBI, with the increase correlating with injury severity, clinical outcomes, and 3‐month mortality.^186^, ^191^, ^194^ Furthermore, the research documented a notable increase in UCH‐L1 levels in the serum of patients experiencing mild‐to‐moderate levels of TBI. In these patients, UCH‐L1 levels can be detected within serum immediately following trauma and are linked to GCS scores, CT‐identified lesions, and neurological intervention measures.^149^, ^183^, ^186^, ^194^, ^195^ These discoveries underscore the significant role of UCH‐L1 not only in moderate‐to‐severe TBI but also in its potential functioning as a marker for TBI with mild‐to‐moderate intensity.^194^, ^196^, ^197^

Besides being a biomarker of TBI, UCH‐L1 may serve as a crucial factor in other nervous system diseases. For instance, it serves as an indication of neuronal cell loss following an aneurysm‐induced subarachnoid hemorrhage^198^ and indicates disrupted BBB functionality following severe injury.^199^ In addition, after severe TBI accompanied by a viral infection, UCH‐L1 levels in both CSF and serum increase, with deceased patients typically exhibiting higher levels compared to survivors.^186^, ^189^ Finally, a study on newborns showed that after moderate‐to‐severe hypoxic–ischemic encephalopathy, the concentration of serum UCH‐L1 also increased.^200^ This discovery further expands the potential application. Variations in UCH‐L1 levels serve as a marker for a range of diseases of the nervous system.

##### Predict the prognosis

When considering UCH‐L1 as a prognostic marker for clinical outcomes, studies have demonstrated that the measurement of UCH‐L1 level within 24 h after admission is closely related to long‐term clinical results. Among pediatric patients with moderate‐to‐critical TBI, UCH‐L1 levels increased substantially, but they did not increase appreciably after mTBI. This indicates that UCH‐L1 could serve as a valuable indicator for assessing the extent of TBI in children and predicting their long‐term clinical prognosis.^149^ This discovery underscores the potential of UCH‐L1 in evaluating TBI severity and predicting long‐term prognosis. Subsequently, the extended study found that UCH‐L1, serving as a predictor of adverse clinical outcomes, had a specificity of 100% and a sensitivity of 27%. This confirms that UCH‐L1 concentrations are directly linked to the extent of injury, further establishing its role as a TBI biomarker.^201^ In addition, the study shows that integrating biomarkers such as UCH‐L1 into the established prognosis model can significantly improve the predictive value of functional prognosis after TBI.^201^ These findings support the notion that circulating biomarkers offer additional predictive worth for functional recovery after TBI by incorporating biomarkers, notably UCH‐L1, into existing prognostic frameworks.^202^ This integration not only helps doctors better understand patients' conditions and prognoses but also guides them to make more personalized and effective treatment plans. Lastly, an analysis of the biodynamic behavior of UCH‐L1 in the serum and CSF of individuals with severe TBI further corroborated previous data, confirming that UCH‐L1 is a potential biomarker for critical head trauma.^186^, ^194^ A study examining the biodynamic characteristics of UCH‐L1 levels in the blood serum and CSF of patients suffering from severe acute TBI reported similar results.

#### 4.2.3 Future research direction

As research into the mechanisms of UCH‐L1 in TBI deepens, the potential diagnostic and therapeutic applications of UCH‐L1 in TBI may be further explored in the future. For example, a specific detection method based on UCH‐L1 can be developed to enhance its ability to accurately detect and distinguish TBI diagnosis; the combined use of UCH‐L1 and other biomarkers can also be studied to evaluate TBI more accurately.

### 4.3 Glial fibrillary acidic protein

#### 4.3.1 Introduction of GFAP

In the study of TBI biomarkers, GFAP ranks second in terms of extensive study, following S100‐β. Both GFAP and the application of GFAP‐BDP as a diagnostic tool for acute TBI have been investigated, demonstrating considerable promise as candidates for enhancing the diagnosis, classification, and targeted treatment of TBI patients.^203^, ^204^ GFAP was first isolated and extracted from the brain tissue of multiple sclerosis patients in 1969 and was then described as “plaque protein.”^185^ In 1999, for the first time, GFAP was accurately quantified in human blood, revealing heightened serum levels in 12 of 25 patients diagnosed with critical TBI.^205^ Intriguingly, following its discovery, GFAP was determined to be a vital element in cases of fibrous gliosis, which is distinguished by the presence of fibrous astrocytes and neurons that have lost their myelin sheaths. Since then, it has undergone intensive research as a biomarker with both diagnostic and prognostic applications in TBI.^206^

GFAP serves as the primary intermediate filament astrocytic protein, a macroglial cell specific to the vertebrate CNS. It performs functions such as cell‐to‐cell signaling, controlling mitosis, and ensuring the BBB remains intact.^207^ It maintains the structural integrity and mechanical resilience of glial cell cytoskeletons.^205^, ^208^ Damage to astrocytes triggers the production of GFAP.^187^, ^209^, ^210^, ^211^ Subsequently, an elevation in GFAP expression points to CNS recovery efforts following cellular damage or demise.^209^, ^211^ Damage to the astrocyte cytoskeleton triggers the leakage of GFAP into the blood circulation.^187^, ^209^ After brain injury, stroke, and various neurodegenerative disorders, the presence of GFAP in the bloodstream has been documented.^187^ Underscoring its unique specificity to the CNS, coupled with its detectability in blood after TBI, has prompted researchers to explore the practicality of utilizing GFAP to serve as a marker for TBI.^187^, ^209^, ^210^, ^211^ These studies are expected to provide new methods and tools for early diagnosis, disease monitoring, and treatment effect evaluation of TBI.

#### 4.3.2 The role of GFAP in TBI

##### Early diagnosis value

GFAP stands out as a brain biomarker due to its robust specificity to the brain and high expression levels in brain tissue, making it a crucial tool for investigating and diagnosing brain disorders. The United States witnesses an alarmingly high incidence of TBI exceeding 1.7 million cases annually.^212^ Among these, TBI caused by shock and explosion overpressure waves is particularly common among military personnel and has been a common injury in recent wars.^213^, ^214^ The latest research shows that the GFAP level in patients' blood increases significantly within 24 h after a TBI virus infection. This finding helps to reduce unnecessary CT scans, and brain injury can be evaluated more accurately by detecting GFAP levels. This detection method has proven to be effective in treating patients with varying degrees of TBI, from mild to severe, and may offer advantages over another frequently used biomarker, S‐100β, especially for patients with mild TBI.^215^ GFAP's ability to detect rapid increases in blood concentration shortly after TBI makes it suitable for early TBI diagnosis. Compared to traditional diagnostic methods such as CT scanning, GFAP detection has the advantages of rapidity and convenience.

##### Assess the severity of the disease

In the setting of both penetrating TBI and brain injuries induced by overpressure blast waves in rats, consistent findings from studies indicate a notable elevation of GFAP concentrations in CSF and/or serum within 4 to 24 h postinjury.^216^, ^217^ Although studies have described the serum GFAP spectrum of critical and moderate TBI, there are still few special studies on serum GFAP levels after mild TBI.^184^, ^205^, ^218^, ^219^, ^220^ Numerous studies have conclusively demonstrated a connection between elevated levels of GFAP in the serum and both the severity of the trauma and resultant health outcomes in patients with critical TBI.^177^, ^203^, ^218^, ^221^ Increased blood concentrations of GFAP have been demonstrated to forecast cerebral hypoxia, a secondary injury that manifests after brain trauma in severe TBI patients,^222^ in samples of serum and plasma collected following TBI of mild‐to‐moderate severity.^223^ The value of GFAP as an indicator of brain injury has been recognized in individuals with mild‐to‐moderate TBI.^224^ After severe TBI, notable increases in GFAP levels have been detected within the CSF and the blood components of serum and plasma within a period of 3 to 34 h.^177^, ^185^, ^203^, ^225^, ^226^ As early as 1999, research already indicated an elevation in serum GFAP levels among patients with severe head trauma.^205^ More contemporary studies have further validated GFAP as a definitive indicator of CNS damage,^203^, ^218^, ^219^ showcasing its potential as a valuable biomarker for anticipating clinical outcomes.^144^, ^187^, ^203^, ^218^, ^226^

##### Predict the prognosis

A recent study has underscored the greater sensitivity and selectivity of serum GFAP measurements in comparison to serum S‐100β and NSE in TBI cases.^227^ Additionally, it has been established that serum GFAP concentrations exhibit a significant prognostic value for predicting mortality and overall outcome.^218^ When measured employing a prototype point‐of‐care platform test, GFAP emerges as a highly responsive blood‐borne biomarker, showing exceptional diagnostic accuracy in predicting brain trauma on CT scans in individuals with TBI. Notably, GFAP surpasses S‐100β as a diagnostic biomarker of TBI, especially in cases with positive head CT scans.^228^, ^229^ The findings of the research advocate for broadening the existing indication for GFAP to encompass more critically injured individuals and a testing period extending up to 24 h posttrauma.^206^ The recognition of GFAP's diagnostic capabilities by the FDA has led to its approval for use in evaluating intracranial injuries based on its impressive predictive accuracy for CT scan results.^215^ Furthermore, research has indicated that elevated GFAP levels in those experiencing mTBI are linked to abnormal brain imaging results, which may suggest the need for surgical intervention. This biomarker can also differentiate between patients involved in motor vehicle accidents with orthopedic injuries and those with mTBI.^224^, ^230^ These discoveries emphasize the versatility and clinical importance of GFAP as a diagnostic tool in managing TBI.

##### The role of GFAP‐BDP in TBI

GFAP‐BDPs, in addition to GFAP levels, have garnered considerable clinical interest. As a biomarker, GFAP‐BDP possesses crucial characteristics necessary to better forecast radiographically apparent injuries in all severities of TBI, including accuracy, discriminative power, calibration, and clinical applicability. Notably, GFAP‐BDP levels have demonstrated the ability to effectively differentiate the severity of intracranial injuries, even when considered apart from other established injury predictors.^223^ Research has demonstrated that, within 1 h after the injury, individuals with mild‐to‐moderate TBI exhibit elevated levels of serum GFAP‐BDP. This elevation is associated with the presence of intracranial lesions and the requirement for neurosurgical intervention.^231^ Research on GFAP‐BDPs in severe TBI has garnered significant attention, with these biomarkers being associated with injury severity, intracranial lesions, and mortality.^203^, ^204^ These studies have further confirmed the presence of GFAP‐BDPs across the full spectrum of TBI severities, ranging from severe to mild‐to‐moderate cases, reinforcing their potential as indicators for assessing brain injury.^184^, ^224^ Collectively, these findings highlight the potential of GFAP‐BDP to emerge as a hopeful early sign of intracranial injury. Consequently, GFAP‐BDP serves as a beneficial supplement to current TBI diagnostic methods, providing clinicians with an additional tool for enhanced patient assessment and management of TBI.

#### 4.3.3 Application prospect of GFAP in TBI treatment

##### Therapeutic target

GFAP may become a potential target for TBI treatment in the future because of its key role in the pathophysiological process of TBI. By regulating the expression or function of GFAP, the reaction of astrocytes can be influenced, thus alleviating brain injury and promoting nerve repair.

##### Drug development

At present, some drug development research for GFAP is underway. These drugs are designed to regulate the response of astrocytes by inhibiting or promoting the expression of GFAP, with the ultimate goal of treating TBI.

### 4.4 Joint detection of GFAP and UCH‐L1

GFAP and UCH‐L1 stand out as two important indicators of brain damage and hold a pivotal role in diagnosing, assessing the severity, and predicting the prognosis of TBI. Combined detection of GFAP and UCH‐L1 can provide a more comprehensive reflection of the pathophysiological processes involved in brain injury, thereby enhancing the sensitivity and specificity of the diagnosis.

#### 4.4.1 Advantages of joint detection of GFAP and UCH‐L1

##### Improve the sensitivity and specificity of diagnosis

Although UCHL‐1 and GFAP each exhibit notable potential as prognostic and diagnostic markers for TBI, several studies have evaluated their combined use, revealing that this approach enhances the accuracy and precision in identifying TBI.^185^, ^209^, ^232^, ^233^ Combined analysis of UCH‐L1 and GFAP concentrations in blood serum has shown remarkable effectiveness in differentiating TBI patients from healthy individuals, providing superior sensitivity and specificity when compared to individual biomarkers. A case–control study observed significant elevations in the serum concentrations of UCH‐L1 and GFAP in individuals with severe TBI, providing valuable insights into the severity of the injury and subsequent outcomes. This research underscored a robust link between serum biomarker elevations and GCS ratings, as well as CT imaging findings, with marked increases in GFAP concentrations in patients presenting with mass lesions and higher UCH‐L1 levels in those with diffuse injury patterns.^177^, ^185^ The pivotal ALERT‐TBI study underscores the remarkable sensitivity of GFAP and UCH‐L1 biomarker concentrations in blood, assessed within the first 12 h postinjury, in determining the necessity of CT imaging. The use of GFAP and UCH‐L1 biomarkers has the potential to reduce the frequency of unnecessary head CT scans in cases of acute TBI by up to one‐third while maintaining an exceptionally low false‐negative rate. The clinical application of GFAP and UCH‐L1 biomarker detection offers several advantages, including reduced radiation exposure and cost associated with CT scans.^234^

##### Evaluate the condition more comprehensively

At the beginning of 2018, the FDA approved the clinical application involving GFAP and UCH‐L1 levels in adults with a diagnosis of TBI, enabling healthcare providers to determine the need for a CT scan within the crucial 12‐h window following injury.^235^, ^236^, ^237^ The authorization for these biomarkers is particularly significant due to the time‐critical nature of TBI assessment and the imperative need to identify patients with intracranial traumatic hemorrhage before clinical deterioration. The measurement of care points using GFAP and UCH‐L1 provides a practical and effective method for rapid evaluation and appropriate treatment interventions. The combined deployment of both GFAP and UCH‐L1 has demonstrated remarkable precision in anticipating the occurrence of intracranial abnormalities among individuals with mTBI. This capability is indispensable in directing clinical decision‐making and minimizing unnecessary CT scans and their associated radiation exposure.^238^ A pilot study involving mTBI patients found that the combining UCH‐L1 and GFAP biomarkers with cutting‐edge MRI imaging procedures bolstered the precision in diagnosing injuries. This indicates that the combination of biomarkers and imaging modalities can bolster our capacity to detect and characterize TBI, especially in its initial stages.^239^ Furthermore, on January 8, 2021, the FDA approved the i‐STAT TBI test, which can simultaneously measure the levels of UCH‐L1 and GFAP, and can be used to evaluate the TBI of adult patients.^240^ UCH‐L1 enhances the diagnostic value of GFAP by measuring distinct molecular events occurring in varying cell types. This complementary aspect of the two biomarkers facilitates a more thorough evaluation of brain injury and improves diagnostic precision.^136^

##### Predict prognosis

An additional monitoring study disclosed a connection between GFAP and UCH‐L1 levels, proposing that a combined examination of these biomarkers can yield more effective information for diagnosing and evaluating the prognosis of TBI compared to individual analyses.^209^ The report highlighted the correlation between plasma concentrations of GFAP and UCH‐L1 and the degree of TBI within the initial week following injury, providing further evidence for the utility of these biomarkers in diagnosing TBI in its acute stage. This discovery highlights the importance of early biomarker measurement in guiding clinical decision‐making and ensuring timely and appropriate treatment intervention.^241^ A study found that the combined analysis of UCH‐L1 and GFAP levels enabled precise distinction of diffuse injury from local injury at admission. In particular, the ratio between these two proteins can predict the prognosis better than any biomarker alone. This highlights the importance of considering both neuronal and glial injuries when evaluating TBI because it provides a more comprehensive understanding of injuries and their potential consequences.^185^

#### 4.4.2 Significance of glial neuronal ratio

The concept of the glial neuronal ratio (GNR) introduces a fresh perspective in distinguishing focal from diffuse injuries in TBI. Through the analysis of the ratio between GFAP and UCH‐L1 levels, GNR offers insights into the relative degree of damage to glial cells and neurons, shedding light on the main cell group impacted by the injury.^185^ Though GNR can't directly reflect the severity of injury, it complements absolute biomarker concentrations, which are more reliable in evaluating injury severity. Initial biomarker concentrations, particularly GFAP and UCH‐L1, play a vital role in evaluating the severity of the immediate trauma and categorizing the injury stage. This distinction is vital for directing clinical decision‐making and guaranteeing suitable therapeutic interventions.^185^ Studies have shown that GFAP and UCH‐L1 exhibit distinct patterns of release following TBI. GFAP always performs well in detecting mTBI and CT lesions and predicting the necessity of neurosurgery within 7 days. In contrast, UCH‐L1 is most useful in the early stage after injury.^242^ These findings have important clinical implications for healthcare professionals, as they provide valuable information on the optimal timing for biomarker measurement after trauma.^204^, ^224^ Additionally, the GFAP and UCH‐L1 may identify children at risk of adverse outcomes and provide therapeutic and diagnostic benefits in clinical nursing and research.^201^

#### 4.4.3 Significance of joint detection

The joint detection of UCH‐L1 and GFAP provides a more comprehensive assessment of brain injury and offers stronger support for clinical decision‐making. By leveraging the complementary strengths of these biomarkers, healthcare professionals can gain a more nuanced understanding of the injury and develop more targeted treatment plans.

### 4.5 S‐100β protein

The protein S‐100β holds a pivotal role in TBI, functioning as a vital indicator for diagnosing TBI, evaluating its severity, and predicting its prognosis. As a well‐researched and clinically significant indicator, S‐100β occupies a central position in the management of TBI, with a long history of research supporting its use in assessing brain injury and predicting outcomes. Its unique properties and widespread expression in the CNS make it an invaluable tool for clinicians and researchers alike.

#### 4.5.1 Overview of S‐100β protein

S‐100β, a protein that binds calcium, is primarily produced in astrocytes and Schwann cells, with a high concentration in white matter. Released by astrocytes and other glial cells within the CNS, as a biomarker of protein formation of TBI, it has been widely studied.^243^, ^244^ In 1965, S‐100β was named due to its unique solubility of 100% in saturated ammonium sulfate solution. The homodimer composed of β‐chains, possessing a molecular mass of 10.5 kDa, is prevalent in the brain, especially in the cytoplasm of astrocytes.^245^, ^246^, ^247^ In addition to its expression in diverse cell types, S‐100β exhibits numerous localized regulatory functions, encompassing the regulation of cellular division, growth, programmed cell death, energy production, and inflammatory responses.^248^ In 1995, the first study on human TBI evaluated S‐100β as a serum biomarker for assessing brain damage. Given that S‐100β is predominantly expressed and localized primarily within the CNS and exhibits an increase in serum concentration correlated with brain damage, it is regarded as a promising biomarker for assessing brain damage.^249^ Since then, S‐100β has been the most extensively studied brain injury biomarker, with many studies focusing on its role as a marker of astrocyte injury or death.^250^, ^251^ Furthermore, S‐100β has been established as a robust and independent outcome predictor in TBI.^252^

#### 4.5.2 The role of S‐100β protein in TBI

##### Assess the condition of TBI

S‐100β is recognized as an indicator of astrocyte damage or demise in the context of TBI. Consistently, various studies have reported a correlation between serum levels of S‐100β and both GCS scores and neuroradiological findings at hospital admission.^253^, ^254^, ^255^, ^256^ Furthermore, two separate research teams have uncovered that serum levels of S‐100β can effectively distinguish between patients with mild and severe TBI.^244^, ^257^ Additionally, a substantial body of evidence supporting an association between serum S‐100β concentrations and patient outcomes has been observed. It is noteworthy that multiple investigations have indicated that elevated S‐100β levels are linked to more severe injuries and poorer prognoses.^218^, ^253^, ^255^, ^258^, ^259^

In 2012, a research report pointed out that S‐100β can be used as a biomarker for differential diagnosis, which can be used to distinguish local injury from diffuse injury in patients with TBI.^260^ With intracranial hypertension, multiple research endeavors have revealed a strong association between S‐100β concentrations and intracranial pressure (ICP) readings. A study conducted in 2005 revealed a strong link between the maximum serum S‐100β concentration and the ICP, suggesting that changes in S‐100β levels may be closely associated with increased ICP due to brain injury, thereby reinforcing S‐100β's value as a brain injury biomarker.^256^ A 1998 study found that patients with ICP below 25 mmHg had lower S‐100β serum levels compared to those with ICP of 25 mmHg or more, indicating a possible relationship between S‐100β levels and ICP increases, further emphasizing its potential as a biomarker for brain injury or other ICP‐elevating brain disorders.^218^ The study in 2005 further confirmed that a direct relationship was observed between the highest level of S‐100β in CSF and the ICP value. This means that with the increase in ICP, the concentration of S‐100β in CSF will also increase accordingly. This discovery not only enhances the understanding of S‐100β serving as an indicator of brain damage or other intracranial lesions but also provides a theoretical foundation for assessing changes in ICP through S‐100β level monitoring.^261^ Regarding the timing of S‐100β protein peaks in severe TBI patients, studies have shown different kinetic patterns in serum and urine. For example, the serum level peaks within 6 h and then gradually drops to 96 h, whereas the urine level drops within 48 h and then rises again at 96 h.^262^ However, it's crucial to note that extracranial injuries can influence S‐100β levels, and they may only occasionally increase after mild TBI. Moreover, normative S‐100β concentrations are relatively high in young children, making it a less‐reliable screening tool for detecting intracranial pathology in this age group.^263^, ^264^, ^265^, ^266^, ^267^ Despite certain limitations, such as a lack of specificity, S‐100β protein is still regarded as a valuable biomarker for predicting intracranial hypertension, particularly in the initial stages of TBI.^268^ In summary, S‐100β is a well studied and clinically relevant TBI biomarker closely related to the severity of injury, intracranial hypertension, and patient outcomes. Although it has some limitations, especially in detecting mild TBI and intracranial pathology in young children, S‐100β continues to be an essential tool for clinicians in assessing and managing TBI patients.

##### Predict prognosis

The concentration of S‐100β protein can predict normal imaging results in individuals with mTBI, thereby serving as a valuable tool in reducing unnecessary CT scans for this patient population. In Scandinavia, the management guidelines for adult head injuries advocate for the utilization of S‐100β protein throughout the initial period of treatment for minor, mild, and moderately severe TBIs.^90^, ^269^ A robust connection between S‐100β levels and CT scan results has been established through research. Studies have shown a notable correlation between the extent of contusions observable on CT scans and the concentration of S‐100β in the serum.^253^ Additionally, a subsequent study conducted by the same research team discovered a significant relationship between S‐100β values and CT results.^256^ However, it is crucial to acknowledge that S‐100β is present in various tissues outside the brain, and its levels may elevate due to factors unrelated to brain injury. For example, extracranial injuries and physical exercise have been shown to elevate S‐100β levels.^270^, ^271^ Therefore, when interpreting S‐100β levels, it is important to consider these potential confounding factors.

#### 4.5.3 Conclusion

In conclusion, the S100β protein, serving as a neurobiomarker, has exhibited considerable significance in assessing, predicting the prognosis, and making a diagnosis for TBI. Monitoring changes in serum S‐100β protein levels can provide clinicians with a more accurate and reliable basis for diagnosis and prognosis evaluation. Nevertheless, it is vital to take into account that various factors, including age, gender, and pre‐existing health status, can influence the variation in S‐100β protein levels. Therefore, when interpreting S‐100β protein levels, it is crucial to thoroughly consider the patient's individual circumstances and comprehensive clinical history.

### 4.6 Neurofilament proteins

#### 4.6.1 Basic characteristics of NF protein

The existence of NF in the cytoplasm of neurons was first observed by neuroanatomists Ramón y Cajal and Golgi using silver staining 100 years ago, and their remarkable abundance in axons was noted. After decades of electron microscope analysis, it was revealed that NF in axons was a bundle of filaments with a diameter of 10 nanometers, and these filaments were named “neurofilaments” (NFs) because they only existed in neurons.^270^ NF is a unique component of neurons, primarily functioning in preserving the shape and size of neurons, as well as facilitating the conduction of nerve impulses along axons.^272^ An elevation in the concentration of NF protein in the CSF serves as an indicator of axonal damage. Given NF's pivotal role in promoting axon integrity and support, its increased levels can be observed following axonal injury.^273^

NFs, the primary constituents of the neuronal cytoskeleton, comprise mainly three subcomponents: NF‐H, NF‐M, and NF‐L. These subunits collectively form NFs within neurons, performing a critical function in supporting neuronal architecture and activity.^274^ Although all proteins fall under the category of NF, each possesses distinct characteristics and is released in varying circumstances. Proposed as the primary contributor, axonal white matter damage has been implicated in influencing outcomes after both mild and severe TBIs.^275^ NF‐L exhibits the highest concentration in large‐diameter myelinated axons that extend into deeper regions of the brain and the spinal cord.^276^ Notably, NF‐L is predominantly found in large‐diameter myelinated axons that project into deeper regions of the brain and the spinal cord.^277^, ^278^ NF‐L, along with the subunits NF‐M and NF‐H, serves as a crucial scaffolding protein within the neural cytoskeleton, playing vital roles in the branching and growth of axons and dendrites.^279^ Among various biomarkers, NF‐L stands out as a promising indicator of disease severity in conditions, including multiple sclerosis and amyotrophic lateral sclerosis, as well as in both minor and critical instances of TBI.^273^ In the context of concussive TBI, serum NF‐L emerges as a sensitive and dynamic biomarker for axonal injury. This marker holds potential for detecting and monitoring CNS injury in concussions.^280^

#### 4.6.2 The role of NF in TBI

##### Reflect neuronal damage

The report highlighted the significant effectiveness of serum NF‐H measurements in predicting the type of injury and prognosis of TBIs in children. Specifically, it was noted that children with DAI had notably higher serum NF‐H levels during the initial CT scan.^281^ Another study compared serum NF‐H concentrations between patients with DAI and those with focal injuries, revealing that within 10 days of admission, the median serum NF‐H level in DAI patients was substantially higher than in those with focal TBIs.^260^ These findings underscore NF‐H's potential as a biomarker for differentiating DAI from focal injuries. Numerous studies have established NF‐L as a significant biomarker for nervous system disorders characterized by axonal damage or degeneration, encompassing mild TBI.^282^, ^283^, ^284^, ^285^ Moreover, serum NF‐L levels have been observed to elevate in American football players throughout the season, as well as in individuals with TBI.^286^, ^287^, ^288^ This aligns with recent discoveries identifying NF‐L as a significant protein indicator for distinguishing between abnormal and normal CT scans results.^289^ Conversely, our results suggest that serum NF‐L exhibits superior diagnostic and prognostic values compared to GFAP, tau, and UCH‐L1 in the scenarios of subacute and prolonged TBI.^290^ For patients with TBI, serum NF‐L concentrations assessed within 48 h after the injury have been shown to differentiate between those with abnormal CT findings and those with normal CT images.^276^ Furthermore, in athletes' concussions, it is found that even if athletes have no obvious symptoms, the increase in NFL level still suggests potential nerve damage.^291^

##### Evaluate the severity and prognosis of the disease

Measurement of NF‐L in CSF of boxers after a bout has established a connection with the severity of brain impairment.^245^ In adult American football players, increased NF‐L concentrations correlate with TBI, with elevated levels acting as a marker of axonal injury.^286^ Similarly, elevated levels of NF‐M are observed in CSF and blood serum samples collected from adult patients with severe TBIs.^292^ Research revealed a relationship between the elevated levels of NF and adverse outcomes or mortality in patients with TBI.^144^ Furthermore, increased levels of pNF‐H in the CSF of amateur athletes post‐bout have been observed,^288^ and pNF‐H also appears to predict fatality following brain trauma in pediatric patients.^281^ Conversely, existing studies indicate that months to years after injury, serum NF‐L levels can distinguish between individuals with mild, moderate, and severe TBI, as well as distinguish them from healthy controls.^293^, ^294^, ^295^ Serum NF‐L holds promise as a diagnostic tool for acute and frequent sports‐related head injuries and individuals experiencing subacute and chronic stages of TBI.^296^ In the setting of neurodegenerative disorders, the level of NF‐L in CSF is established as a sensitive biomarker of neuroaxonal damage.^273^ For TBIs, measuring NF‐L in CSF has demonstrated prognostic value for both mTBIs and sTBIs. Nevertheless, due to the invasive procedure required for lumbar punctures to obtain CSF, routine and repeated lumbar punctures may not be feasible in clinical practice for TBI patients, making blood‐based biomarkers a more favorable option.^278^ The extent of NF‐L increase is more pronounced than that of T‐tau protein, suggesting that mTBI has a greater impact on long myelinated fibers in white matter versus short unmyelinated axons located in the cortex. Until now, CSF levels of NF‐L have emerged as the most sensitive fluid‐based biomarker for detecting axonal damage.^278^, ^283^ Collectively, these studies imply that a single episode of TBI may cause long‐term axonal degeneration, which can be identified in serum through the use of NF‐L as a biomarker, extending from months to years' postinjury.^297^

### 4.7 Tauopathy biomarkers

#### 4.7.1 The basic overview of tau

In 1975, Kirschner's laboratory discovered a kind of protein named “tau,” which is the abbreviation of “tubulin‐associated unit,” and was named after its ability to induce microtubule formation.^298^ In 1985, the immunohistochemical evidence of tau protein in paired spiral filaments (PHF) was first reported; this discovery provides an important clue for understanding the role of tau protein in neural degenerative disease.^299^ After this, in 1986, researchers discovered that the characteristic neurofibrillary tangles (NFTs) observed in post‐mortem brain tissues from individuals with AD were composed of highly phosphorylated tau (p‐tau) protein. This revelation underscored the significant role of tau protein in the pathological process of AD and presented a novel perspective on the disease's pathogenesis.^300^, ^301^ Further, in 1991, a characteristic distribution pattern of tau protein–related neurofibrils was identified in the brains of individuals with AD, prompting the establishment of a staging system named after these findings.^302^ This staging system revealed that the temporal and spatial spread of tau protein was a contributing factor to the advancement and severity of the disease.

Tau occupies a central position in brain function through its binding to tubulin, thereby facilitating the polymerization and stabilization of microtubules, particularly those found in the extended neuronal processes of the brain.^298^, ^303^, ^304^, ^305^ Tau, a protein associated with neuronal microtubules, is prevalent in cortical axons lacking myelin^245^, ^299^, ^306^ and is a structural element of the axon cytoskeleton in both the CNS and peripheral nervous system (PNS).^307^ In typical physiological states, p‐tau regulates its biological activity.^308^, ^309^ Aggregates of p‐tau form NFTs, which are recognized as the pathological signature of tauopathies, comprising AD and chronic traumatic encephalopathy, frontal‐temporal dementia (FTD), and other related conditions.^310^, ^311^, ^312^ Within the brain, tau primarily functions to bind tubulin, thereby enhancing the polymerization and stabilization of microtubules, particularly those located in the elongated neuronal axons.^303^, ^304^, ^305^

#### 4.7.2 The role of tau in TBI

##### Screening and diagnosis

Although there is no screening or diagnosis method of tauopathy biomarker directly for TBI, examining the dynamic alterations of tau protein following TBI may assist in pinpointing individuals who face an elevated risk of developing subsequent neurodegeneration. Epidemiological research suggests that TBI serves as a predisposing factor for tau‐related disorders.^309^, ^313^, ^314^, ^315^ P‐tau and NFTs become detectable within the first 6 h following TBI.^316^, ^317^ The level of NFT was increased in about one‐third of postmortem individuals; those who survive moderate‐to‐severe TBI demonstrate a link between tau protein aggregation and a single instance of TBI.^318^

##### Prediction of disease progression

By monitoring the tau protein level in CSF or blood of patients after TBI, it may be possible to predict the risk of cognitive decline or the progress of neurodegeneration. The increase in tau protein concentration is a sign of axonal injury, which has been confirmed in observations after concussion and other head injuries.^305^, ^307^

##### Clinical trial

When evaluating therapeutic strategies for neurodegeneration following TBI, alterations in tau protein concentrations can function as an indicator of therapeutic efficacy. Research has focused on T‐tau, p‐tau, and the p‐tau/T‐tau ratio as potential indicators of TBI. In the context of TBI, physical strain, proteolytic fragmentation by calpain and caspase enzymes, and the triggering of calcium‐dependent kinase activity cause the release of tau protein from microtubules, leading to reduced microtubule affinity and augmented tau phosphorylation.^319^, ^320^ The concentration of tau protein derived from the brain in serum may prove to be an effective biomarker for distinguishing the extent of TBI and overseeing clinical progress on the injury day and at the 7‐day postinjury mark.^321^ Previous studies on TBI have found an elevation of tau in CSF specimens collected acutely from patients with moderate‐to‐severe TBI.^75^, ^320^, ^322^ Research has shown that when measuring blood (serum or plasma) samples, the current T‐tau determination method will simultaneously quantify CNS and peripheral tau protein.^323^, ^324^

The research shows that when the level of tau protein reaches or exceeds 114.5 pg/mL using receiver operating characteristic (ROC) curve analysis, its sensitivity and specificity in predicting adverse results are 88% and 94%, respectively. This result strongly suggests that the serum tau protein level may be an effective index to forecast the outcome for patients suffering from severe TBI.^325^ Research conducted on a sample of 39 individuals with severe TBI revealed that the baseline T‐tau concentration in CSF exhibited a strong correlation with outcomes measured after 1 year. There is a significant correlation between the initial T‐tau level in CSF and Glasgow Outcome Scale‐Extended (GOSE), which indicates that the tau protein level may be an important index to predict the prognosis of patients. In addition, the investigation revealed a notable distinction in outcomes among patients who passed away and those who recovered when CSF T‐tau levels surpassed 2126 pg/mL 2–3 days after the traumatic event. The accuracy and precision of this marker in assessing fatality likelihood exhibited 100% and 81%, respectively, further underscoring the pivotal role of utilizing tau protein to anticipate the future health status of individuals suffering from severe TBI.^326^ In the prognosis prediction of individuals diagnosed with severe TBI, the initial cut tau protein (c‐tau) level showed extremely high sensitivity and specificity, which were 92% and 94%, respectively.^327^ Research conducted on individuals experiencing mild TBI, like amateur boxers, revealed a rise in the amount of T‐tau protein in CSF collected through lumbar puncture between 4 and 10 days postmatch. Notably, even boxers who were not knocked down demonstrated similar increases.^326^, ^327^, ^328^ If boxers do not participate in further competitions, their CSF T‐tau protein levels return to normal within 8–12 weeks postcompetition. Therefore, CSF T‐tau protein levels may serve as a marker for axonal injury in gray matter neurons.^278^, ^283^ In addition, compared to the preseason baseline, the plasma tau protein level of athletes increased within a few hours after concussion.^283^, ^326^ Elevated levels of serum tau protein are associated with white matter damage and a reduction in fractional anisotropy, which indicate compromised white matter integrity. Furthermore, the concentration of tau protein correlates with the number of blows sustained by athletes experiencing concussions, hinting at its potential as a dose‐dependent biomarker, consistent with the clinical manifestations observed following multiple head traumas.^329^ Preliminary results from the pilot group of the TRACK‐TBI study further indicated that p‐tau protein effectively distinguished chronic TBI from the healthy control cohort, as indicated by the area under the curve (AUC) values for the ROC curve spanned from 0.963 to 1.00.^320^ Although T‐tau remains a key biomarker for TBI studies, phosphorylated tau protein concentrations and the blood‐based p‐tau to T‐tau ratio may offer improved diagnostic and prognostic information, warranting further scrutiny. Studies have shown that the ratio of p‐tau to T‐tau exhibits greater efficacy than T‐tau alone in differentiating between mTBI and sTBI, as well as in distinguishing between positive and negative findings on skull CT. The importance of the p‐tau/T‐tau ratio serving as a diagnostic indicator and predicting long‐term outcomes in both acute and chronic TBI is highlighted.^330^

Although the application of tauopathy biomarkers in TBI is still in the exploratory stage, studying the dynamic changes of tau protein after TBI may offer fresh insights into the mechanisms underlying secondary neurodegeneration and present potential biomarkers for the creation of novel diagnostic, prognostic, and treatment methodologies. More interdisciplinary cooperation and in‐depth research are needed to promote the development of this field in the future.

### 4.8 Myelin basic protein

#### 4.8.1 The basic overview of MBP

MBP, a protein found in oligodendrocytes, serves as a vital structural element of the multilayer myelin sheath that envelops nerve fibers, which is essential for normal myelination and axonal signal transduction and plays an adhesion role between the cytoplasm surfaces of a single myelin sheath.^331^ Functioning as an insulator, the myelin covering in the nervous system augments the speed at which axonal impulses travel. MBP sustains the correct formation of the myelin sheath by engaging with the lipids found in the myelin membrane.^332^ Within the myelinated axonal tracts of white matter, the proteolytic breakdown of MBP, for instance, occurs through the action of calpain.^333^, ^334^ Consequently, under these circumstances, MBP or its fragments may be released into biological fluids following TBI. As MBP is predominantly present in the CNS, it emerges as a potential candidate for TBI biomarkers, as indicated in studies.^335^, ^336^ In addition, MBP ranks as the second most prevalent protein within the CNS myelin sheath.^331^ Moreover, it has been identified in CSF of individuals with demyelinating conditions, including multiple sclerosis,^337^ as oligodendrocyte/white matter injury occurs in DAI, which is a characteristic of mTBI.^338^, ^339^

#### 4.8.2 The role of MBP in TBI

MBP serves as a biomarker indicative of myelin sheath damage following TBI, possessing significant value in the diagnostic process of TBI. In cases of minor‐to‐moderately severe TBI, the increase in MBP levels occurs at a gradual pace, with the maximum concentration potentially reaching a peak after 24 h or beyond. In severe TBI, a significant increase can be observed within 12 h after injury, and it quickly reaches its peak. The peak concentration is usually higher because severe TBI involves more extensive myelin sheath damage. By detecting the changes in MBP content in CSF or blood, doctors can facilitate the early detection and evaluation of therapeutic response for TBI. For example, research has demonstrated that MBP is released into CSF after controlled cortical impact (CCI) in rats,^340^ and serum MBP levels also elevate in children with sTBI and adults with mTBI.^340^ In addition, MBP can be used as one of the important indices to gauge the efficacy of therapy and predicted course of TBI. As a marker of penetrating TBI (pTBI), the level of MBP does not increase immediately after injury.^78^, ^341^ On the contrary, it peaks at 48–72 h after injury, which may reflect axonal injury.^78^, ^148^ Research has established that MBP concentration can serve as a predictive indicator of clinical outcomes in children under the age of 3 who have experienced TBI.^146^ In the study of 152 children with TBI, it was found that the initial MBP concentration was intimately tied to the clinical development of children aged 4 and below, but the correlation was relatively weak compared to children aged 4 and above. This discovery reveals the differences and limitations of serum MBP concentration as a prognostic indicator in children of different ages and provides a new perspective and reference for evaluating the prognosis of TBI in children more accurately in clinics.^145^

MBP appears to exhibit high specificity in diagnosing TBI in infants, with a specificity rate of 100%, albeit accompanied by a relatively low sensitivity of only 34%.^342^ In research involving TBI patients, both adult and pediatric, concentrations of MBP in blood serum and CSF have been investigated as a possible indicator for nonaccidental TBI^343^ or pTBI^147^, ^342^ and outcome estimation^148^, ^344^, ^345^ in children. After TBI, the concentration of MBP usually reaches its peak in 48–72 h, and the peak concentration seems to reflect TBI more accurately than the initial concentration.^146^, ^148^ Above all, MBP plays a pivotal role in TBI, and alterations in its stability and content are crucial for assessing the extent of myelin sheath injury, which dictates the progression of the illness, and directing therapeutic strategies.

### 4.9 Inflammatory biomarkers

#### 4.9.1 Inflammatory response and TBI

Inflammation's significance in the evolution of TBI‐associated pathological changes has been a key area of investigation in numerous studies.^346^, ^347^, ^348^ No matter the severity of TBI, the inflammatory reaction always plays an indispensable role in its pathophysiological process.^30^ TBI of varying severities has been linked to distant neurodegeneration, persistent inflammatory responses, and alterations in vascular structures.^34^, ^53^, ^349^, ^350^ Inflammation serves as a crucial element in the aftermath of TBI, addressing the secondary insult to the CNS; the mitigation of inflammation stands out as a potential target for effective treatment.^351^, ^352^ In the inflammatory process within the brain after TBI, several pivotal aspects come into play, such as BBB impairment, edema development, microglia activation, astrocyte activation, and displacement, accompanied by the excretion of inflammatory agents like cytokines, as well as the migration of blood‐derived leukocytes into the brain's substance.^347^ Notably, in granulocytes, particularly neutrophils, which are mobilized soon after the occurrence of TBI, the peripheral circulation becomes instrumental in the early stages of the pathogenic process, enhancing swelling and oxidative stress and triggering the secretion of inflammatory cytokines and neurotoxic proteolytic enzymes.^353^, ^354^ The neuroinflammatory response is regulated by numerous pivotal mediators, including pro‐inflammatory cytokines (such as IL‐1β, IL‐6, TNF‐α), chemokines (such as CCL2, CCL5, CXCL1), secondary signaling molecules (such as NO, prostaglandins), and ROS. These mediators are mostly produced by activated resident CNS cells such as microglia and astrocytes.^355^ In addition, endothelial cells and perivascular macrophages play an important role in the transmission of inflammatory signals in the CNS.^356^

After TBI, neurodegenerative processes may persist for several months.^357^, ^358^ Cytokine‐induced inflammation is instrumental in the emergence of secondary pathologies after brain trauma.^359^ Primary and secondary TBIs elicit inflammatory responses through the activation of inflammatory proteins, particularly cytokines produced by activated microglia and leukocytes.^349^ Numerous inflammatory cytokines have been documented in the literature as potential indicators of TBI. Certain inflammatory cytokines, notably IL‐6, exhibit a sharp increase and remain at elevated levels within 6 h following TBI.^360^ In a research study, including 114 adults suffering from severe conditions, it was observed that an elevation in IL‐6 levels in CSF was linked to unfavorable clinical results 1 year after TBI.^361^ Additionally, a study aimed to determine whether plasma biomarkers such as S‐100β, NSE, SVCAM, SNCAM, IL‐6, and IL‐8 might foretell the onset of fatigue in pediatric individuals with varying degrees of TBI severity.^362^ Reports indicate increased levels of TNF‐α,^363^, ^364^, ^365^ IL‐8,^366^, ^367^, ^368^ and IL‐10^363^, ^366^, ^369^ detected in the CSF and serum levels of patients with TBI. Moreover, the extent of brain injury is related to the plasma protein levels of IL‐6.^369^, ^370^

A multitude of research has emphasized that serum levels of IL‐6, TNF‐α, IL‐2, and IFN‐γ serve as precise biomarkers for assessing brain damage.^371^ Recently, these findings have enhanced the secretion of TNF, IL‐1β, IL‐6, and IFN‐γ proteins, as well as their corresponding gene upregulation, in postmortem human brain tissue following TBI.^372^ Animal studies simulating brain hypoxia or trauma independently trigger the swift release of inflammatory cytokines, including IL‐1β, IL‐6, and TNF.^373^, ^374^, ^375^ Furthermore, studies using models that mimic focal brain injuries indicate that posttraumatic hypoxia further deteriorates brain tissue damage,^376^, ^377^, ^378^ and intracranial edema,^379^ as well as sensorimotor, behavioral, and cognitive deficits.^380^, ^381^, ^382^

IL‐1β, IL‐4, and IL‐12 are critical mediators of posttraumatic neuroinflammatory responses and correlate with neuronal toxicity and damage to cortical neurons.^261^, ^383^, ^384^, ^385^ Conversely, the upregulation of IL‐6, IL‐8, and IL‐10 expression is indicative of a neuroprotective function.^386^, ^387^ This could be explained by their ability to modulate the production of nerve growth factor and other neurotrophic factors, including Brain‐Derived Neurotrophic Factor (BDNF) and Glial cell line‐Derived Neurotrophic Factor (GDNF), which are derived from the brain and glial cells, respectively, essential for the regeneration of damaged brain neurons.^367^, ^388^, ^389^ As an important index of inflammatory reaction, inflammatory biomarkers are of great significance for evaluating the progress of the disease after TBI, predicting the prognosis and guiding the treatment. Adiponectin and HMGB1 are both inflammatory markers in patients with TBI. Adiponectin predicts adverse outcomes and mortality, and HMGB1 is not only an important predictor of 1‐year mortality but also enters phagocytic microglia after translocation from the nucleus to the cytoplasm, so it is not only a biomarker but also a potential therapeutic target for TBI.^390^, ^391^, ^392^ As a member of the lectin family, galectin‐3 is involved in microglia activation, and its concentration in the plasma of TBI patients increases, which may predict hospital mortality. However, low plasma ficolin 3 level is associated with poor outcomes, and ficolin 3 is an activator of the lectin complement pathway.^393^, ^394^ In addition, the level of mannose‐binding lectin (MBL) in the serum of TBI patients increased.^395^

#### 4.9.2 Clinical application of inflammatory biomarkers

Experimental models, along with clinical research, have evidenced a robust link in the interplay between secondary brain injury and neuroinflammatory processes, predominantly fueled by the activation of T‐cells and monocytes, which subsequently trigger the production of cytokines within the brain.^396^, ^397^ Various pro‐inflammatory and anti‐inflammatory factors exhibit fluctuations following TBI, prompting their investigation as potential biomarkers for diagnosing and predicting TBI.^349^, ^398^ Recent research indicates that even mTBI can trigger an inflammatory response. In adult and immature rats, microarray studies showed that the expressions of cytokines and inflammatory genes were significantly upregulated after TBI.^399^, ^400^ Clinical and experimental studies show that the expression levels of cytokines, including TNF‐α, TGF‐β, IL‐1β, IL‐6, and IL‐10, rise following TBI.^347^, ^401^, ^402^ These cytokines increased in brain tissue, CSF, and serum after TBI.

The quantities of IL‐1β and IL‐6 cytokines, as referenced in Ref. [403], and combinations of S100‐β with either IL‐6 or L‐selectin, as mentioned in Ref. [404], are indicative of the gravity of the injury and predictive of detrimental long‐term consequences. In terms of treatment, preclinical research has shown that blocking these signaling molecules has the potential to mitigate brain damage.^405^ Furthermore, in numerous research endeavors, it has been observed that levels of IL‐10, a cytokine that suppresses IL‐2, increase poststroke or TBI.^406^, ^407^ Cytokines act as traditional signaling molecules to attract more neutrophils and monocytes from the bloodstream to the damaged tissue, thereby initiating the inflammatory response. TBI can trigger neuroinflammation as a secondary response. During the healing phase, glial cells, including microglia and astrocytes, produce a range of pro‐inflammatory cytokines, such as IL‐1β, IL‐6, and TNF‐α. However, these substances may also have neurotoxic consequences.

Post‐TBI, the generation of proteins with anti‐inflammatory properties takes place, involving cytokines such as IL‐6, IL‐8, and IL‐10. Notably, after severe TBI, IL‐10, a cytokine, demonstrates an upregulation in both CSF and serum.^408^, ^409^, ^410^, ^411^ After severe TBI, the amount of NGF present in CSF emerges as a useful indicator of brain damage, especially noting its upregulation as a key sign. Within the initial 48 h following head trauma, a combination of reduced IL‐1β expression and certain patterns in IL‐6 levels is associated with a positive neurological outcome. Additionally, IL‐6 expression levels can provide valuable clinical and prognostic insights.^336^ Both inflammatory and anti‐inflammatory cytokines are reported to undergo alterations due to TBI, and it is believed that their combined effects determine the overall level of inflammation.^346^

Pro‐inflammatory markers, but not anti‐inflammatory markers, are increased in the blood serum of affected individuals.^412^ In patients with isolated TBI, specific inflammatory biomarkers such as IL‐6 have been suggested as possible indicators of elevated ICP.^413^ The findings in these reports suggest that inflammation is a contributing factor to the pathophysiology of severe TBI. The initiation of immune‐inflammatory responses following TBI is facilitated by inflammatory mechanisms. Among the indigenous cells of the CNS, microglia emerge as a likely primary contributor of these inflammatory mediators within the CNS. Experimental investigations focusing on genetic factors in TBI and stroke models have unveiled that inflammatory genes exhibit increased expression during the acute phase following these conditions.^414^, ^415^, ^416^

Prior research has indicated that inflammasome proteins could serve as potentially valuable indicators of the extent and pathological outcomes associated with TBI.^84^ These studies have also highlighted inflammasome‐related proteins that present themselves as promising indicators of TBI‐induced pathological effects.^84^, ^417^, ^418^ A recent multiplex analysis of potential serum biomarkers has identified IL‐6 as a marker for pediatric TBI.^419^ Additionally, IL‐10, an anti‐inflammatory mediator, has exhibited the potential to distinguish between CT‐positive and CT‐negative cases of mTBI.^420^ Research has demonstrated that IL‐10 may serve as a useful indicator to help healthcare professionals anticipate whether mTBI patients will exhibit positive or negative findings on CT scans.^420^ Upon injury to the CNS, various cells release IL‐10, which has demonstrated a protective role by reducing cytokine activity, pro‐inflammatory responses, and apoptotic processes.

Measuring inflammatory biomarkers after sustaining a TBI may serve as a tool for tracking disease progression, diagnosing injuries, and estimating long‐term results. However, several investigations have emphasized that when multiple injuries occur, inflammatory markers may not possess the ability to specifically distinguish brain injury.^413^, ^421^, ^422^ The future role of inflammatory mediators as indicators of injury in TBI remains uncertain, primarily due to their lack of specificity. Nevertheless, advancements such as the establishment of animal models for multitrauma, the utilization of combined marker panels, and novel sampling techniques could potentially resolve this issue. Studies using clinically pertinent animal models of TBI should focus on elucidating both the isolated and collective impacts of therapeutically targeting inflammatory proteins identified as key biomarkers of the post‐TBI inflammatory response, with the aim of improving histopathological and functional recovery.

### 4.10 Extracellular vesicles

#### 4.10.1 The basic overview of EVs

Extracellular vesicles (EVs) are nanoscale to micron‐scale membrane vesicles released by cells, which harbor bioactive compounds, including proteins, lipids, and nucleic acids. These vesicles have a vital impact on intercellular communication, material exchange, and signal transmission. According to their origin and characteristics, EVs can be divided into many types, encompassing exosomes with a diameter of 30–100 nm, microvesicles (MVs) ranging from 100–1000 nm, and apoptotic bodies (ABs) measuring 2–to 5 μm.^423^, ^424^ These EVs have sparked significant interest as they present novel possibilities for identifying and directing the management of TBI. EVs are membrane‐enclosed sacs released by cells into the extracellular space, functioning as a means of intercellular communication by transporting cargo, including DNAs, RNAs, and proteins, between cells.^425^, ^426^ Secreted by diverse cell types into bodily fluids, EVs participate in intercellular communication.^424^ EVs are excreted by all brain cell types and are typically enclosed within a lipid bilayer membrane and decorated with distinctive surface markers and harboring intraluminal DNA, RNA, proteins, and metabolites, which function as indicators of glial cell and neuronal health. They are crucial factors in the pathological process and regenerative processes following CNS injuries.^427^, ^428^, ^429^ Robust evidence suggests that EVs can originate from CNS cells^430^, ^431^, ^432^ and have a crucial function in governing and adjusting neuroinflammation, neuronal reconstruction, and neurite extension.^433^, ^434^

The active participation of EVs during the progression of TBI, along with their display of distinct surface markers indicative of brain cell types,^427^, ^428^, ^435^ makes them valuable potential biomarkers. At the same time, EVs' characteristics, including their immune responsiveness, stability, extended half‐life, and ability to cross the BBB,^436^ make them excellent candidates for delivering therapeutic agents. Another category of EVs utilized for identifying TBI is MVs. These are also known as microparticles, originating from the outward budding of the plasma membrane and ranging in size from 100 nm to 1 μm. The membranes of MVs are composed of lipid microdomains that include cholesterol, phospholipids, and receptors, displaying a variety of shapes.^437^, ^438^ Studies have reported that MVs contribute to the initiation and development of injuries resulting from TBI.^439^ Notably, patients with severe TBI exhibit elevated levels of microparticles compared to healthy controls.^440^

#### 4.10.2 Exosomes

In 1983, Pan and Johnstone made the groundbreaking discovery of exosomes when they observed the release of transferrin receptors, linked to small 50‐nm vesicles, which are released from maturing reticulocytes into the extracellular space through a process involving receptor‐mediated endocytosis and recycling.^441^ Over the years, the field of EV research has advanced significantly. Exosomes, specifically, are characterized as nanosized EVs with a diameter ranging from 30 to 150 nm. They are produced within cells and are expelled when multivesicular endosomes merge with the cell membrane.^442^, ^443^ Exosomes are composed of a bilayer lipid membrane that encapsulates a variety of biomolecules within their interior, which contain mRNAs, miRNAs,^444^ lipids,^445^ and proteins.^446^ Accumulating evidence points to exosomes as key mediators of intercellular communication, as they convey their contents to recipient cells to adjust the functions of the target cell.^447^ Exosomes have been demonstrated to readily penetrate the BBB, enter the systemic circulation, and show elevated levels across diverse neurological conditions.^448^ Many studies have confirmed that brain‐targeted drug carriers (such as exosomes) show significant neuroprotective effects, and their mechanism lies in delivering antioxidants to neurons, thus effectively reducing the level of ROS.^449^, ^450^

A body of evidence from various studies has highlighted exosomes' capability to serve as a valuable indicator for both diagnosis and prognosis in TBI. Exosome excretion following TBI could facilitate the elimination of surplus cellular constituents arising from pathological events or tissue harm due to the injury.^451^, ^452^ Emerging data support the notion that EVs/exosomes govern neuroinflammation, synaptic flexibility, and neurovascular integrity while adjusting BBB permeability, thereby affecting cellular responses to brain trauma.^453^ An investigative analysis comparing exosome profiles in the CSF of TBI patients and healthy individuals highlighted the importance of the ratio of p‐tau to T‐tau, which is indicative of both diagnosing and predicting long‐term outcomes of acute and chronic TBIs.^193^ Exosomes can be released by many kinds of cells, including astrocytes.^454^ Astrocytes are abundant glial cells in the CNS, and their derived exosomes (AS‐Exos) can effectively inhibit the oxidative stress that leads to neuronal apoptosis.

In this context, although it is generally believed that the concentrations of circulating markers primarily reflect brain injury, multiple investigations have suggested that the production and release of exosomes are modulated by diverse microenvironmental factors, including oxidative stress, elevated levels of pro‐inflammatory cytokines, and hypoxic conditions, which can trigger the release of proteins from compromised neurons and astrocytes,^455^, ^456^ all of which are characteristic of acute brain tissue following severe TBI. Therefore, it is reasonable to hypothesize that exosomes secreted after acute TBI might serve as a conduit for the disposal and export of excess cellular debris—residuals of the pathological process or injury resulting from the disease.^451^, ^452^ These effects may exert widespread pleiotropic regulatory impacts and additionally aid in neuroplastic changes and tissue restoration after injury. These exosomes encapsulate a diverse array of bioactive molecules, encompassing miRNAs, messenger RNAs (mRNAs), and proteins such as cytokines; these elements engage in cell‐to‐cell signaling under both physiological and pathological circumstances.^457^, ^458^

#### 4.10.3 MicroRNA

EVs are characterized by a lipid bilayer membrane that encapsulates a variety of cargo, including proteins and miRNAs.^459^, ^460^ MiRNAs, being small noncoding RNA molecules, are considered essential regulators of gene expression and epigenetic alterations.^459^ Stem cell–originated EVs have been identified as vehicles for nucleic acids, facilitating the reprograming of hematopoietic progenitor cells through the horizontal transfer of mRNAs.^461^ Recent studies have shown that miRNA is implicated in the development of various diseases, such as TBI, spinal cord injury, and tendon injury.^462^, ^463^, ^464^

##### The basic overview of miRNA

MiRNA stands as a significant nucleic acid component within EVs and holds promise as an innovative biofluid biomarker for neurotrauma. Belonging to a vast family, miRNAs are small and endogenous; additionally, these are ubiquitously conserved noncoding RNA species that function at the posttranscriptional stage, affecting protein synthesis and gene expression patterns.^465^ These noncoding RNAs consist of 19–28 nucleotides and have a pivotal function in controlling the production of proteins in both neurons and glial cells at the posttranslational level. Furthermore, miRNAs are instrumental in regulating protein expression and synaptic maturation.^465^ At the same time, miRNA has been suggested as a potential therapeutic target for numerous diseases, including TBI.^466^, ^467^

##### The research progress of miRNA in TBI

TBI elicits a multitude of molecular and biochemical changes in the CNS, involving modifications in mRNA and protein expression, disturbances in signal transduction pathways, alterations in cell proliferation rates, apoptosis, and neural regeneration.^468^, ^469^, ^470^ miRNAs are invaluable in TBI research due to their abundant presence within the CNS, where approximately 70% of all miRNAs are present in both the CNS and the PNS. A key advantage of using miRNAs as biomarkers for TBI can traverse biofluids with enzymatic degradation prevented, permitting their detection within the first hour following an injury. A restricted number of investigations have examined serum miRNA profiles as indicators for TBI.^100^, ^471^, ^472^, ^473^ In the year 2009, several studies focusing on expression profiling underscored the possible significance of miRNA in TBI.^474^, ^475^ Microarray profiling of miRNA expression levels following TBI offered a means to simultaneously analyze multiple targets, providing a comprehensive evaluation of relative expression changes and identifying a potential miRNA expression signature for TBI. It has been reported that compared to other organs, the brain shows an increased abundance and heightened expression levels of miRNAs.^476^ The results showed that the expression levels of several miRNAs changed significantly in the hippocampus after TBI. These miRNAs may affect many biological processes and functions involved in the pathophysiology of TBI by regulating the expression of their target protein.^475^

Among TBI patients, miR‐320c stands out as a promising biomarker candidate. Alterations in miR‐320c levels have been observed in the blood plasma of adult individuals suffering from severe TBI and the saliva of pediatric patients experiencing mTBI.^472^, ^477^, ^478^ Furthermore, this miRNA demonstrates temporal fluctuations within the CSF of patients following the occurrence of severe TBI, offering an approximation of the length of TBI‐related symptoms. Another intriguing biomarker, miR‐92a, emerges as a candidate for TBI diagnosis. Specifically, serum miR‐92a levels are observed to be upregulated in patients with mTBI, as well as in those with severe TBI; higher miR‐92a levels are detected in both CSF and serum.^471^, ^472^ An upregulation of miR‐92a has been observed during the acute and subacute phases postinjury. Research indicates that elevated levels of this upregulated miR‐92a inhibit angiogenesis after ischemic events.^479^, ^480^ Consequently, the prompt upregulation of miR‐92a may hold particular importance in patients with severe TBI, particularly those who also have intracranial hemorrhage (ICH) or neurovascular comorbidities. When comparing plasma miRNA profiles of patients, researchers discovered a marked reduction in miR‐16 and miR‐92a levels during the first 24–48 h following injury in patients with severe TBI compared to healthy controls, whereas the level of miR‐765 increased significantly during this period. These changes are considered indicative of severe TBI, suggesting that these three miRNAs have potential value in distinguishing injury types associated with intracranial hypertension and may serve as candidate biomarkers for clinical diagnosis and differential diagnosis.^481^ In‐depth studies have shown that by inhibiting the overexpression of Ptgs2 and miR‐212‐5p, iron death can be effectively alleviated, and the learning ability and spatial memory of TBI mice can be significantly improved.^466^ In addition, some studies have revealed that the interaction between astrocytes and neurons through the EV‐miR‐382‐5p‐OPA1 signaling pathway plays a key role in the apoptosis of TBI‐related neurons. The plasma level of EV‐miR‐382‐5p significantly increased in the TBI mouse model and human patients. Inhibition of miRNA‐382‐5p through RVG‐EV‐mediated delivery can effectively protect neurons from TBI. The results show that EV‐miRNA‐382‐5p not only has a clinical diagnostic value but also shows the potential of treating TBI.^482^ It is found that miR‐21, as a new typical miRNA, regulates many signal pathways such as inflammation, neuronal apoptosis, glial hyperplasia, BBB destruction, angiogenesis, and recovery induced by physical exercise in TBI.^483^ miR‐124 is highly expressed in the brain. As a regulator, miR‐124 participates in cell apoptosis and proliferation, closely related to the pathophysiological development of TBI, and plays a role by interacting with various biomolecules and signal pathways.^484^

Considering the wide array of miRNAs that display distinctive expression profiles within specific brain regions and cell types, moreover, considering their consistent stability and abundance in various biofluids, miRNAs offer great promise as outstanding indicators of TBI. Although the research on miRNA in TBI is still in the basic stage, its potential in TBI treatment has been widely discussed. In the future, with the deepening of research and the continuous development of technology, miRNA is expected to become one of the important means of TBI treatment.

### 4.11 Other emerging biomarkers

#### 4.11.1 αII‐spectrin protein

##### The basic overview of the αII‐spectrin protein

The αII‐spectrin protein constitutes a component of the axolemmal cytoskeleton, where it anchors to ankyrin. This interaction is crucial for connecting axolemmal components to the presynaptic terminal.^485^, ^486^ The spectrin protein is crucial for maintaining the stability of the nodal and paranodal architecture in myelinated axons.^486^ Predominantly localized in neurons, αII‐spectrin exhibits a high abundance in axons and presynaptic regions.^487^ It functions as a vital substrate for the enzymes calpain‐1 and calpain‐2, which are implicated in cellular necrosis, and caspase‐3, which is involved in apoptotic processes.^488^

##### The degradation of αII‐spectrin in TBI

The cortical membrane cytoskeleton is predominantly composed of αII‐spectrin, which functions as a primary target for the cysteine proteases, specifically calpain and caspase‐3. Extensive evidence suggests that αII‐spectrin undergoes processing by these proteases, resulting in characteristic cleavage products in vivo following experimental TBI.^489^ Primarily localized in neurons and concentrated in axons and presynaptic regions, αII‐spectrin undergoes degradation into fragments known as Spectrin Breakdown Products (SBDPs), with molecular weights of 145, 150, and 120 kDa. Notably, it is worth noting that SBDP120 is produced through caspase‐3‐induced proteolytic cleavage and is primarily associated with the process of apoptosis. During ischemia or TBI, calpain and caspase‐3 serve as key players in inducing necrotic and apoptotic cell death, respectively.^179^, ^340^, ^490^, ^491^

##### The application of αII‐spectrin decomposition products as cell death markers

Multiple studies involving adult patients have shown increased concentrations of spectrin SBDPs in CSF following severe TBI.^179^, ^180^, ^481^ Elevated CSF SBDP145 levels have also been observed in rat models of controlled cortical impact.^78^, ^490^ Recently, SBDPs have emerged as potential biomarkers for detecting brain injury in rats and humans.^178^, ^179^, ^491^ A prospective, controlled study revealed that measuring αII‐spectrin degradation in CSF could function as a reliable indicator of severe TBI in human subjects.^179^ This study focused on variations in 150 and 145 kDa BDPs, primarily produced by calpain, and 120‐kDa SBDPs generated by caspase‐3. Notably, calpain proteolysis, although primarily linked to oncotic necrosis, is not exclusive to this process, whereas caspase‐3 proteolysis is chiefly associated with apoptosis.^127^ Recent investigations have highlighted C‐terminal SBDPs of αII‐spectrin, specifically SBDP150 and SBDP145 formed by calpain during necrotic processes, and SBDP120 created by caspase‐3 during apoptotic events, as potential indicators of cell death in TBI animal models, human CSF samples, and neuronal cell culture injury models in vitro.^180^, ^490^, ^492^, ^493^, ^494^ These results provide robust evidence for the utilization of α‐spectrin proteolytic fragments as markers of ongoing secondary proteolytic cell death mechanisms in individuals with brain injury.^127^ Assessing the levels of SBDPs, each possessing distinct features, could offer crucial prognostic insights during the initial assessment of patients with severe TBI, and function as an innovative element in early outcome prediction and risk evaluation.^180^ Moreover, ample evidence suggests the presence of SBDPs in preclinical mouse models of TBI, human TBI investigations, and studies on subarachnoid hemorrhage.^495^, ^496^, ^497^ Although our research primarily focuses on the analysis of SBDP levels in CSF, the findings imply that spectrin fragments originating from both calpain and caspase activities may potentially function as biomarkers for TBI.^179^, ^489^, ^498^ In conclusion, SBDPs, and notably CSF SBDP 150, emerge as a promising candidate for a distinguishing diagnostic indicator, possessing the ability to discern focal versus diffuse injuries in the acute phase of TBI.

In recent years, significant progress has been made in investigating αII‐spectrin as a biomarker of cell death in TBI. Nevertheless, several obstacles and concerns remain, including developing strategies to enhance the sensitivity and specificity of detection techniques and clarifying the accurate relationship between changes in decomposition product levels and the pathophysiological progression of TBI. These problems need further research and exploration in the future.

#### 4.11.2 Metabolomics

##### Introduction to metabolomics

The application of metabolomics in the field of TBI is a new and important research direction, which provides us with a new way to deeply understand the pathophysiological mechanism of TBI, evaluate the extent of injury, and predict the outcome. The concept of metabolomics emerged from research conducted during the 1980s and 1990s.^499^ Metabolomics is the field dedicated to the investigation of the metabolome, encompassing the quantitative assessment of all metabolites present within a biological system.^500^ Metabolomics offers a holistic method for studying the composition, roles, and exchanges of low‐molecular‐weight metabolites across cellular, tissue, and biofluid boundaries.^501^ This promising branch of research on brain injury biomarkers is of particular significance, given that a neurometabolic crisis is a hallmark feature of concussion.^502^ The objective of metabolomics research is to distinguish pathological metabolic signatures from normal physiological ones and predict classification labels utilizing a distinct set of metabolic indicators.^503^, ^504^

##### Application of TBI metabolomics

Increasing emphasis has been placed on small molecules, particularly metabolites, as candidate biomarkers for TBI stratification. Indeed, circulating polar metabolites have demonstrated promising diagnostic and prognostic capabilities for TBI,^505^, ^506^, ^507^ corresponding with imaging results,^508^, ^509^ injury severity, and outcomes 6 months after the injury.^505^ TBI is linked to alterations in the metabolome. As opposed to classical proteomic biomarkers, the amounts of circulating metabolites such as neuronal amino acid (NAA), myoinositol, glycerophospholipids, and choline empower researchers to examine neurometabolism following a TBI event.^510^

NAA undergoes synthesis and is subsequently hydrolyzed in oligodendrocytes to facilitate myelin formation, acts as an indicator of brain injury through its direct association with glucose metabolism, as observed in research conducted in animal models and through human studies utilizing magnetic resonance spectroscopic techniques, as detailed in Ref. [511]. A comprehensive examination of magnetic resonance spectroscopy investigations in individuals with sports‐related concussions indicated that 9 out of 11 studies detecting decreases in NAA after injury met the inclusion criteria, aligning with findings from rodent TBI models.^512^ The data suggest that circulating metabolites are linked to the extent of TBI and may aid in better‐forecasting patient outcomes. Notably, specific lipids, such as lysophosphatidylcholines, ether phosphatidylcholines, and sphingomyelins, are strongly and specifically associated with TBI severity and rank among the most powerful predictors of patient prognosis.^513^ There is a continuing unmet clinical demand for the discovery of cost‐effective and noninvasive biomarkers in TBI, where circulating metabolites present a promising avenue for exploration.^507^, ^514^ Human studies have demonstrated that metabolites are associated with TBI severity, can predict patient outcomes,^505^ and aid in identifying patients requiring CT scans.^508^ Myoinositol, a polyol compound that is prevalent in glial cells, especially astrocytes, functions in membrane phospholipid metabolism and osmoregulation.^515^ Studies have demonstrated an elevation in brain levels after injury in adults.^516^ Moreover, they exhibit increased levels in the occipital cortex of pediatric TBI patients 1 week following injury, with notably higher concentrations noted in patients who experienced adverse outcomes as opposed to those with favorable outcomes.^517^ Our results further demonstrate that metabolites can serve as biomarkers to differentiate between various findings observed in head CT scans. We successfully demonstrated that both lipids and polar metabolites exhibit potential as biomarkers for the clinical diagnosis and prognosis of TBI, including mild forms.

With ongoing technological advancements and in‐depth research, the application prospects of metabonomics in TBI are expected to expand significantly. In the future, more metabolites are expected to be identified to aid in the diagnosis, prognosis, and therapeutic intervention of TBI. In the meantime, metabonomics will also be combined with other genomics technologies (such as genomics and proteomics) to form a new model of multigenomics joint analysis, which will provide more comprehensive and in‐depth information for TBI research. The main biomarker profiles associated with TBI are summarized in Table 1.

**TABLE 1:** Biomarker profiles of TBI.

Biomarker	Primary cellular source	Typical change post‐TBI	Clinical implications
NSE	Neurons	It increased significantly in the first 6 h after injury and reached the peak at 72 h	It was negatively correlated with GCS score and predicted the degree of early neuronal injury
UCH‐L1	Neurons	It increased significantly within 6–24 h after injury	Early neuronal damage markers to predict secondary circulatory shock
GFAP	Astrocytes	It reaches the peak within 24 h after TBI and is directly related to the degree of injury	It can effectively assist in the diagnosis and predict the prognosis related to CT/MRI injury, with high specificity
S‐100β	Astrocytes Oligodendrocytes	The dada peak value increased rapidly within 1–6 h, and the increase was positively correlated with the severity of brain injury	Combined GCS score and imaging examination can significantly improve the degree of injury and early prognosis
NF	Neurons	The levels of NF‐L and NF‐H increased rapidly within 24 h after injury, and the abnormality in the chronic phase suggested that axons continued to degenerate	Distinguish between patients with abnormal CT findings and patients with normal CT images
Tau	Neurons	Tau(p‐tau) can be detected within 24 h in the acute phase and 6 h in the chronic phase	Predicting the risk of tau lesions is related to chronic cognitive impairment
MBP	Oligodendrocyte	Mild and moderate TBI reached the peak at 24 h, and severe TBI reached the peak at 12 h after injury. Abnormalities in the chronic phase suggested demyelinating lesions	Associated with the risk of white matter atrophy and neurodegeneration
Inflammatory	Microglia/astrocytes	TNF‐α reached its peak within 24 h; IL‐6 was expressed in a biphasic manner, and the chronic phase continued with low inflammation	IL‐10 showed CT positive and CT negative of mTBI, and IL‐6 can evaluate the degree of secondary nerve injury and guide anti‐inflammatory intervention
EVs/exosomes	All nerve cells secrete	Within 48 h after injury, exosomes can effectively inhibit neuroinflammation and improve cognitive dysfunction. TBI itself will trigger the abnormal release of endogenous EVs, leading to the pathological spread of tau protein and the diffusion of inflammatory factors	A blood–brain barrier–penetrating carrier, which can be used for therapeutic delivery
miRNA	Neurons	Specific miRNA profiles are related to the stage of injury	As a multitarget regulatory molecule, it can predict the nerve regeneration potential
αII‐spectrin	Neurons	SBDP120/150 increased within 6 h, which was related to calpain activation	The severity of acute injury is related to secondary calcium overload
Metabolomics	Neuron/glial cell metabolism	Glycolysis is enhanced in the acute phase, and mitochondrial dysfunction occurs in the chronic phase	The concentration of metabolites is a good diagnosis and prognosis for TBI, which is related to the imaging results and the degree of injury

Abbreviations: CT, computed tomography; GFAP, glial fibrillary acidic protein; GCS, Glasgow Coma Scale; EVs, extracellular vesicles; IL‐6, interleukin‐6; IL‐10, interleukin‐10; MBP, myelin basic protein; miRNA, microRNA; MRI, magnetic resonance imaging; mTBI, mild traumatic brain injury; NF, neurofilament; NSE, neuron‐specific enolase; p‐tau, phosphorylated tau; ROS, reactive oxygen species; SBDP, spectrin breakdown products; S‐100β, S100 calcium‐binding protein B; tau, tubulin‐associated unit; TNF‐α, tumor necrosis factor‐ α; UCH‐L1, ubiquitin C‐terminal hydrolase L1.

## 5 FUTURE RESEARCH DIRECTION OF TBI BIOMARKERS

The research on TBI biomarkers can be divided into three stages: single protein marker period (20th century), multimarker combination research period (2000–2015), and systematic medicine integration period (2015–present). At present, the research on TBI biomarkers is in the stage of rapid development. Between 2023 and 2025, a large number of discoveries emerged regarding the integration of multiomics, dynamic monitoring, and artificial intelligence applications.^518^, ^519^, ^520^ Balancing classical research with the latest progress is the key to improving the quality of TBI biomarker review. The basic research on traditional markers such as GFAP and UCH‐L1 is important, but the breakthrough discoveries in the past 3 years should be paid more attention.

### 5.1 Pay attention to TBI's more complex structural damage

It is necessary to pay attention to more complex or larger structural damage (such as brain damage) and its influence on the function of NSE. Trauma and brain injury in TBI have different pathological characteristics, so it is necessary to study their biomarker changes separately. With the in‐depth exploration of complex brain structures such as the cerebellum, midbrain, and brain, we are increasingly recognizing the need for further exploration and clarification of the injury impacts of other glial cell types and their diagnostic significance in TBI. Research has indicated that there are notable variations in the permeability of the BBB to various peptides derived from the bloodstream, which may be crucial in modulating the effects of these peptides on the central CNS, especially in the field of TBI related to the cerebellum, midbrain, and brain.^521^

### 5.2 Exploration and verification of new markers

Further exploration of new biomarkers, especially the exploration and verification of new biomarkers by protein genomics, metabonomics, and genomics, is of great significance to deeply understand the pathophysiological mechanism of TBI and promote its diagnosis and treatment.^3^ Protein omics occupies a pivotal position in TBI research due to its abundant presence, significance, and high accuracy. Through deep excavation and comparative analysis of protein data, researchers can screen out new protein markers closely related to TBI. The expression changes in these markers after trauma not only reflect the stress response and degree of injury of the body but also provide important diagnostic clues for doctors.^152^ In addition, the protein's omics technique helps reveal the interaction and pathway between proteins, which provides a new perspective and thinking for formulating targeted treatment strategies. Metabonomics technology focuses on the changes of metabolites in organisms. By measuring the concentrations of metabolites in biological specimens such as blood and urine, researchers can reveal the metabolic regulation mechanism under TBI conditions. The changes in these metabolites often directly reflect the metabolic disorder and injury of the body, which provide important information for understanding the pathological process of TBI. At the same time, metabonomics technology is also helpful in discovering new metabolic pathways and targets and provides new directions and ideas for drug research and development.^522^ Genomics technology focuses on the variation and expression differences of organism genomes.^523^ In the study of TBI, researchers can dig out genetic markers related to TBI by in‐depth analysis of gene sequencing data. The expression changes in these markers not only reflect the gene regulation mechanism of the body but also reveal the relationship between the degree of injury and gene expression, which provides a solid theoretical basis for precision medicine.

### 5.3 Multidomain integration and clinical application expansion

In the medical field, interdisciplinary cooperation is of great significance for the research and treatment of complex diseases, particularly in the investigation of biomarkers for TBI.^3^ The key to promoting the progress of TBI treatment lies in multifield integration and clinical application expansion. Through the integration of neuroscience, material science, artificial intelligence, and other technologies, accurate diagnosis, personalized treatment, and rehabilitation programs can be realized, and the quality and efficiency of medical services can be improved. Some studies have conducted a single‐center prospective longitudinal cohort study, including patients with severe craniocerebral trauma, to explore the role of GFAP in monitoring the changes of illness and evaluating the response to treatment, and to emphasize the importance of interdisciplinary cooperation for an in‐depth study of GFAP.^524^ Future research directions include exploring the application potential of new biomaterials and artificial intelligence in TBI.^525^ Interdisciplinary cooperation, policy, and financial support are important factors to promote technological innovation and clinical transformation in this field. There will be more biomarkers and clinical platforms in the future, and the results need to be coordinated across platforms. The efficiency of health care will benefit from the diversity of providers.^3^ Interdisciplinary cooperation integrates knowledge and technology in many fields, such as medicine, biology, chemistry, and physics, breaks the boundaries of traditional disciplines, promotes in‐depth exchanges and cooperation, accelerates the discovery of new biomarkers and treatment methods, and improves the level of diagnosis and treatment of diseases.^526^ TBI biomarkers have obvious advantages in early diagnosis and disease monitoring, which can detect and intervene in early symptoms of diseases in time, reflect disease progress and treatment effect, and provide a strong basis for doctors.^527^ Based on these research, the development of targeted drugs and treatment programs is an important way to achieve precision medical care, which can improve the treatment effect and reduce the burden on patients. Therefore, it is of great significance to combine the advantages of neuroscience, bioinformatics, and clinical medicine to jointly promote the development of TBI biomarker research. Moreover, the CBI‐M multidimensional classification system (clinical‐biomarker‐image‐regulator) proposed by *The Lancet Neurology* in 2025 marks the paradigm shift of TBI diagnosis from a single scale to precision medicine.^528^ By integrating four dimensions of data, including the GCS‐P scoring system, GFAP/UCH‐L1/S100β triple detection in the acute phase, AI‐enhanced CT/MRI feature extraction, socioeconomic status, and other PSD scale parameters, comprehensive coverage can be achieved from emergency care to rehabilitation. Its technical verification has been supported by clinical demonstration such as the intelligent imaging platform for acute stroke in 2025, and it will be extended to the field of neurodegenerative disease monitoring in the long term.^519^

## 6 CONCLUSION

Current evidence suggests that biomarkers may become important tools for TBI diagnosis. By detecting the level of biomarkers in the blood, CSF, or other body fluids, the existence, severity, and possible prognosis of brain injury can be quickly and noninvasively evaluated. This helps doctors to make treatment plans in time and reduce the pain and complications of patients. The study of TBI biomarkers does show important potential, but it must be clearly recognized that these biomarkers have not yet become the main means of clinical diagnosis. Moreover, biomarkers face the practical obstacles of interlaboratory variability and clinical transformation, and the detection difference between different laboratories is an important factor affecting the clinical application of biomarkers. The transformation of biomarkers from research to clinical practice faces multiple practical challenges, including timeliness, instant detection in operation, interpretation of test results, and a series of other problems. We should fully realize the current technical limitations and obstacles to clinical transformation and avoid overinterpretation of research results.

With the deepening of the research on TBI biomarkers, more biomarkers with higher sensitivity and specificity may be found in the future. At the same time, the continuous progress of technology, such as the application of high‐throughput sequencing and protein omics, will contribute to a deeper comprehension of the pathophysiological mechanisms underlying TBI, thereby furnishing a more precise foundation for diagnosing and treating TBI.

Concurrently, as advances are made in biomarker detection techniques, our comprehension of TBI can be further expanded beyond reliance on subjective symptom reports. Aggregated evidence underscores the potential of TBI biomarkers to serve as a promising diagnostic and prognostic aid, with the potential to ultimately optimize patient treatment and management approaches. Nonetheless, the availability of reliable and specific biomarkers for clinical application is presently restricted and necessitates extensive research efforts. Another hurdle in the realm of TBI biomarkers lies in the fact that numerous ones, although encompassing S100‐β, UCH‐L1, NSE, amino acids, and a host of other markers, are not specific to the brain. These markers can also be found in various other organs and cell types, encompassing the endocrine system, endothelial cells, smooth muscle cells, red blood cells, and peripheral nerves,^529^ which hinder their effectiveness as TBI biomarkers in cases involving multiple traumas.^404^, ^530^, ^531^ In these circumstances, serum biomarkers that are specific to the brain, such as NF‐L, NF‐H, and GFAP, might exhibit superior diagnostic capabilities. The incorporation of these biomarkers into routine clinical practice could aid in diagnosing TBI and enhancing management strategies across the entire spectrum of injuries.

In summary, there is a scarcity of research examining the differential diagnostic capabilities of biomarkers in TBI, necessitating increased efforts to develop sensitive and dependable biomarkers. The future development needs continuous efforts in technical innovation and clinical verification to truly realize its potential to improve the diagnosis and treatment of TBI. The prospect of TBI biomarkers is promising, and persistent exploration in this domain may exert a profound influence on the clinical handling of cases and the prognosis of patients.

## AUTHOR CONTRIBUTIONS

**Xuting Shen:** Writing – original draft; writing – review and editing. **Si Cheng:** Writing – original draft; writing – review and editing. **Shuyi Chen:** Writing – original draft; writing – review and editing. **Mengyue Wang:** Writing – original draft; writing – review and editing. **Tangying Li:** Writing – original draft; writing – review and editing. **Weicheng Xu:** Writing – original draft; writing – review and editing. **Yifan Zhang:** Writing – original draft; writing – review and editing. **Ping Yuan:** Writing – original draft; writing – review and editing. **Lei Shi:** Writing – original draft; writing – review and editing.

## FUNDING INFORMATION

This work was supported by the Program of Key Research and Development projects in Hainan Province (ZDYF2024SHFZ057), Program of National Natural Science Foundation of China (82370057), the Program of Natural Science Foundation of Shanghai (22ZR1452400).

## CONFLICT OF INTEREST STATEMENT

The authors declare that the research was conducted in the absence of any commercial or financial relationships that could be construed as a potential conflict of interest.

## ETHICS STATEMENT

This article is a review of the published literature and does not involve any new studies with human participants or animals performed by any of the authors. Therefore, ethical approval is not applicable.
